# GPR37 Activation Alleviates Bone Cancer Pain via the Inhibition of Osteoclastogenesis and Neuronal Hyperexcitability

**DOI:** 10.1002/advs.202417367

**Published:** 2025-02-18

**Authors:** Kaiyuan Wang, Yongfang Zhang, Ruichen Shu, Limei Yuan, Huifang Tu, Shengran Wang, Bo Ni, Yi‐Fan Zhang, Changyu Jiang, Yuhui Luo, Yiqing Yin

**Affiliations:** ^1^ Department of Anesthesiology, Tianjin Medical University Cancer Institute and Hospital National Clinical Research Center for Cancer State Key Laboratory of Druggability Evaluation and Systematic Translational Medicine Tianjin's Clinical Research Center for Cancer Tianjin 300060 China; ^2^ Shenzhen University Medical School Shenzhen Guangdong 518060 China; ^3^ Graduate School Tianjin University of Traditional Chinese Medicine Tianjin 301617 China; ^4^ Department of Pain Medicine and Shenzhen Municipal Key Laboratory for Pain Medicine The 6th Affiliated Hospital of Shenzhen University Health Science Center Shenzhen Guangdong 518052 China

**Keywords:** cancer pain, GPR37, neuroimmune modulation, neuroprotectin D1, osteoclastogenesis

## Abstract

Osteolytic bone cancer pain is a primary concern for cancer patients with bone metastasis, and current therapies offer inadequate pain relief. The present study demonstrates that activation of the G protein‐coupled receptor 37 (GPR37) by neuroprotectin D1 (NPD1) or artesunate (ARU) alleviates both acute and persistent pain in multiple mouse models of bone cancer. GPR37 agonists also protect against cancer‐induced bone destruction. Mechanistically, NPD1 or ARU binding to GPR37 in macrophages promotes the release of IL‐10, which further inhibits cancer‐induced osteoclastogenesis. Moreover, direct activation of GPR37 in dorsal root ganglion (DRG) neurons and the spinal dorsal horn reduces action potential firing and the frequency of spontaneous excitatory postsynaptic currents (sEPSCs), thereby suppressing cancer‐induced neuronal hyperexcitability. Importantly, the analgesic and protective effects of NPD1 and ARU are abolished in *Gpr37* ^−/−^ mice, and β‐arrestin 2 is identified as a key mediator in IL‐10 release and neuronal inhibition. In patients with bone metastases, plasma levels of endogenous NPD1 are negatively correlated with both pain intensity and the bone resorption marker CTX‐I. Collectively, these findings highlight GPR37 activation as a potential therapeutic strategy for alleviating bone cancer pain through direct and synergistic inhibition of osteoclastogenesis and neuronal hyperexcitability.

## Graphical abstract

GPR37 activation alleviates bone cancer pain via peripheral and neuronal mechanisms. NPD1 or ARU targeting GPR37 in macrophages promotes IL‐10 release through β‐arrestin 2 recruitment, thereby inhibiting cancer‐induced osteoclastogenesis. Additionally, GPR37 activation in the DRG and SDH reduces the No. of action potentials and the frequency of sEPSCs, suppressing cancer‐induced neuronal hyperexcitability.

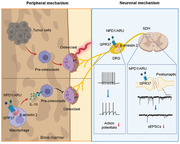

## 1 Introduction

Patients with advanced and metastatic cancer experience varying degrees of pain. Among these, cancer‐related bone pain arises from osteolytic lesions secondary to bone metastasis, as seen in lung and breast cancer, affecting ≈60–84% of patients with advanced tumors.^[^ ^1^, ^2^ ^]^ Cancer pain significantly exacerbates psychological distress, diminishes quality of life, and escalates healthcare expenditures. Moreover, cancer‐induced bone destruction can result in pathological fractures, intensifying cancer pain and impairing overall functionality, thereby increasing tumor‐related mortality. While current clinical treatments such as opioids offer rapid pain relief, they fail to address bone destruction, and research suggests that high opioid doses may exacerbate tumor growth and impair immune surveillance.^[^ ^3^, ^4^ ^]^ Although both radiotherapy and bisphosphonates are effective as tumor‐targeted therapies, they exhibit delayed onset and lack analgesic efficacy for acute breakthrough pain.^[^ ^5^ ^]^ ≈45% of bone cancer pain (BCP) cases remain inadequately controlled.^[^ ^6^ ^]^ Hence, there is an urgent clinical demand for innovative analgesics providing rapid and sustained pain relief while preventing pathological fractures.^[^ ^7^ ^]^

The G protein‐coupled receptor 37 (GPR37), also known as the Parkinson‐related endothelin receptor‐like receptor (Pael‐R), exhibits high expression levels in neurons and glial cells of the brain and is implicated in various neurological disorders such as Parkinson's disease, autism, and stroke.^[^ ^8^, ^9^ ^]^ Recent studies have illustrated that GPR37 is also widely expressed by macrophages and confers protection against infection by bacteria and parasites.^[^ ^10^ ^]^ Neuroprotectin D1 (NPD1), a lipid‐derived mediator, and artesunate (ARU), a semi‐synthetic derivative of artemisinin, have been identified as specific agonists for GPR37.^[^ ^9^ ^]^ NPD1, derived from docosahexaenoic acid (DHA), is known for its potent anti‐inflammatory and neuroprotective properties, particularly in the context of neurodegenerative diseases and injury.^[^ ^11^, ^12^ ^]^ ARU, on the other hand, has been extensively used for its antimalarial effects and has recently been reported to exhibit immunomodulatory, tumor‐suppressive and antidiabetic functions.^[^ ^13^ ^]^ It is reported that GPR37 activation by NPD1 and ARU significantly increases the phagocytic activity of cultured peritoneal macrophages, thereby promoting the resolution of inflammatory pain and infection‐induced pain.^[^ ^14^ ^]^ Furthermore, NPD1 and ARU have been reported to alleviate neuropathic pain and chemotherapy‐induced pain.^[^ ^15^, ^16^ ^]^ However, the role and mechanism of GPR37 in BCP remain unclear. This study aims to investigate the effect of GPR37 agonists on cancer induced bone pain and bone destruction, and further explore the underlying mechanism of GPR37 activation in macrophages and sensory neurons.

## 2 Results

### 2.1 GPR37 Agonists Instantly Alleviate BCP

**Figure 1:**
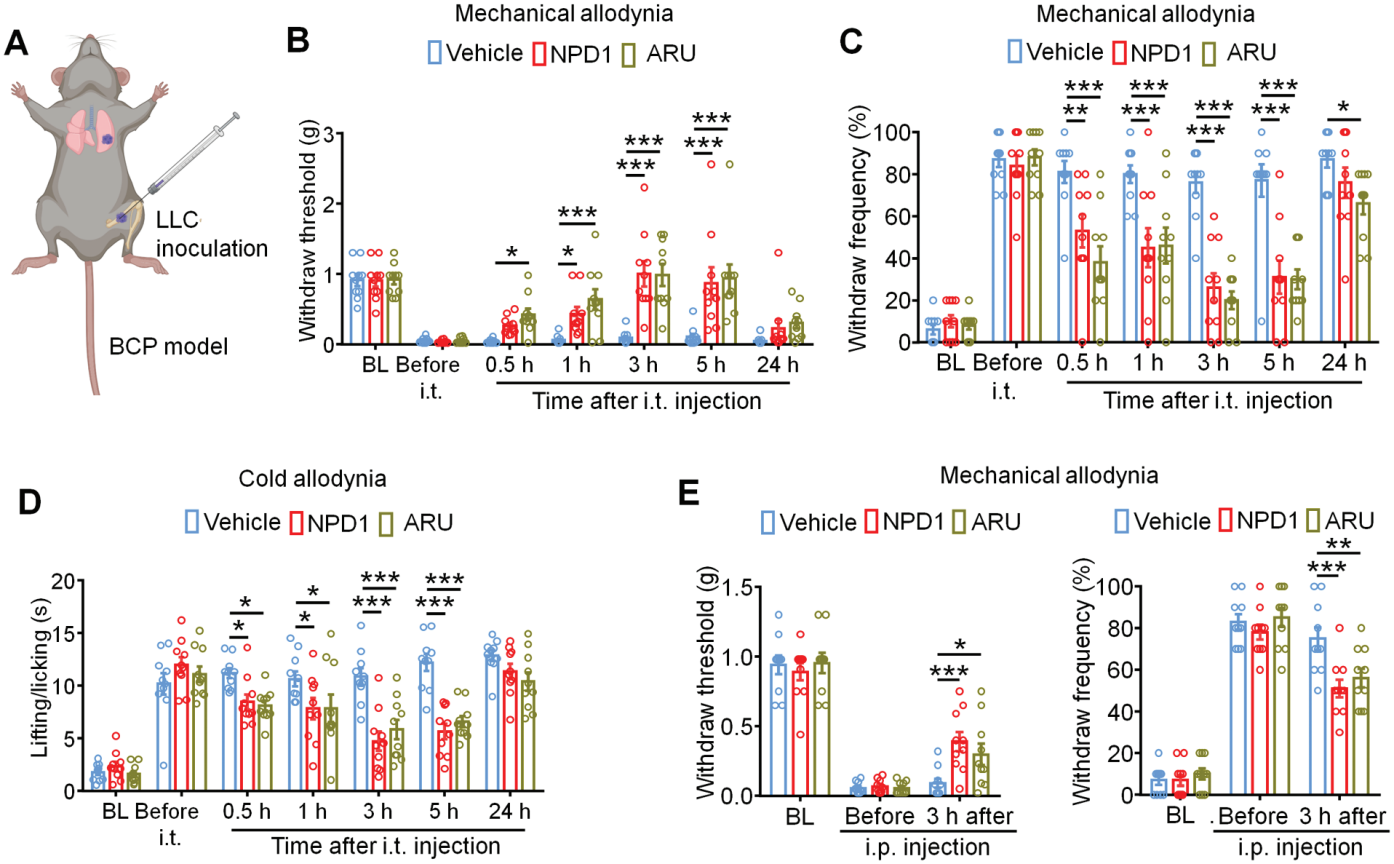
GPR37 agonists instantly attenuate bone cancer pain. A) Schematic of the establishment of bone cancer pain (BCP) model. B,C) Von Fery testing to detect cancer induced mechanical allodynia, as assessed by withdrawal threshold (B) and withdrawal frequency (C) in mice i.t. injected with vehicle, NDP1 (200 ng) or ARU (20 µg) on day 11 after LLC inoculation. n = 10 mice/group. D) Acetone response test to determine cold allodynia after the indicated treatment. n = 10 mice/group. E) Change of mechanical allodynia through von Frey testing after i.p. injection with vehicle, NPD1 (25 µg kg^−1^) or ARU (25 mg kg^−1^) on day 11 after tumor inoculation. n = 10 mice/group. All data are displayed as mean ± SEM, and analyzed using repeated‐measures two‐way ANOVA with Bonferroni's post‐hoc test, *p < 0.05, **p < 0.01, ***p < 0.001.

We first sought to investigate the immediate analgesic effect of GPR37 activation for metastatic cancer‐induced bone pain. C57BL/6 mice were inoculated with the murine lung adenocarcinoma cell line LLC in the left femur to establish a syngeneic BCP model (**Figure** **1**A). On day 11 after tumor implantation, vehicle, NPD1 (200 ng), or ARU (20 µg) were intrathecally injected (i.t.), and behavioral tests were performed on the hindpaw of the tumor‐bearing leg at baseline, before i.t. injection, and at 0.5 , 1, 3, 5, and 24 h after i.t. injection. Von Frey testing showed that GPR37 activation significantly reduced mechanical allodynia at 0.5, 1, 3, and 5 h after i.t. injection (Figure 1B,C). Meanwhile, the acetone response test demonstrated that GPR37 activation alleviated cold allodynia at 0.5, 1, 3, and 5 h after i.t. injection (Figure 1D).

We then determined whether systemic administration of NPD1 and ARU via intraperitoneal injection (i.p.) could lead to pain relief. Results showed that mice receiving a single dose of NPD1 (25 µg kg^−1^) or ARU (25 mg kg^−1^) exhibited attenuated mechanical allodynia at 3 h after i.p. injection (Figure 1E). No apparent sex differences were observed, as the therapeutic effect of NPD1 and ARU on cancer pain existed in both male and female mice (Figure S1A–J, Supporting Information). Taken together, GPR37 activation could instantly alleviate bone pain in the BCP model.

### 2.2 Systemic GPR37 Agonists Produce Long‐Term BCP Relief and Restore Locomotor Function

**Figure 2:**
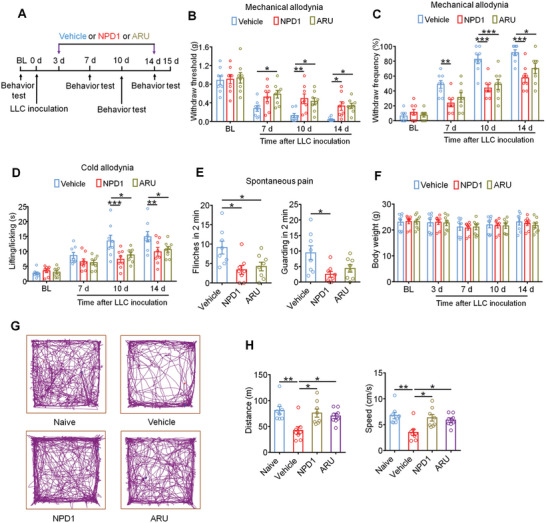
GPR37 agonists attenuate persistent cancer pain and improve locomotor function. A) Experimental design to perform behavior tests after continuous i.p. injection of vehicle, NPD1 (25 µg kg^−1^) or ARU (25 mg kg^−1^). B,C) Von Frey testing to measure mechanical allodynia through withdrawal threshold (B) and withdrawal frequency (C) on day 7, 10 and 14 after tumor inoculation. D) Cold allodynia via acetone response test in mice with indicated treatment. E) Observation of spontaneous pain as shown by flinching (left) or guarding behaviors (right) in vehicle NPD1 or ARU treated mice on day14 after LLC implantation. F) Change of body weight in mice with indicated treatment. G,H) Open field testing detecting distance traveled and mean speed over a 20 min duration in naïve mice or mice with bone cancer pain treated with vehicle, NPD1 or ARU at day 14 after tumor inoculation with representative traces (G) and quantification (H). n = 8 mice/group for the above panels. All data are expressed as mean ±SEM, and analyzed using repeated‐measures two‐way ANOVA with Bonferroni's post‐hoc test (B, C, D, F), or one‐way ANOVA with Bonferroni's post‐hoc test (E, H), *p < 0.05, **p < 0.01, ***p < 0.001.

We next evaluated the long‐term analgesic effect of systemic treatment with GPR37 agonists in a murine BCP model. On day 3 after LLC inoculation, when the tumor became evident, mice were continuously injected intraperitoneally (i.p.) with NPD1 (25 µg kg^−1^) or ARU (25 mg kg^−1^) for 12 days until day 14. Behavioral tests were performed on day 7, 10, and 14 after LLC implantation (**Figure** **2**A). Results showed that NPD1 and ARU treatment significantly attenuated mechanical allodynia by increasing the withdrawal threshold and reducing the withdrawal frequency on day 7, 10, and 14 after tumor inoculation (Figure 2B,C). Mice treated with NPD1 and ARU demonstrated reduced cold allodynia on day 10 and 14 compared with vehicle treatment in acetone response testing (Figure 2D). Spontaneous pain was also detected by measuring flinches and guarding over a 2‐min period on day 14 post‐tumor injection. GPR37 activation treatment significantly attenuated spontaneous and ongoing pain (Figure 2E). Additionally, no differences in body weight were observed among the vehicle, NPD1, or ARU‐treated groups (Figure 2F), indicating that the experimental protocol is relatively safe and without gross systemic gastrointestinal toxicity.

Clinically, patients with metastatic cancer‐induced bone pain often experience diminished mobility, leading to functional impairment and reduced quality of life.^[^ ^17^ ^]^ To determine whether GPR37 activation with NPD1 or ARU could enhance movement activity, we evaluated locomotor function using the open field test. Importantly, mice treated with NPD1 or ARU exhibited increased overall distance of movement and speed of movement on day 14 after tumor inoculation (Figure 2G,H) compared to vehicle treatment. Thus, systemic administration of NPD1 and ARU significantly improved locomotor function in mice with BCP.

### 2.3 GPR37 Agonists Protect Against Cancer Induced Bone Destruction

**Figure 3:**
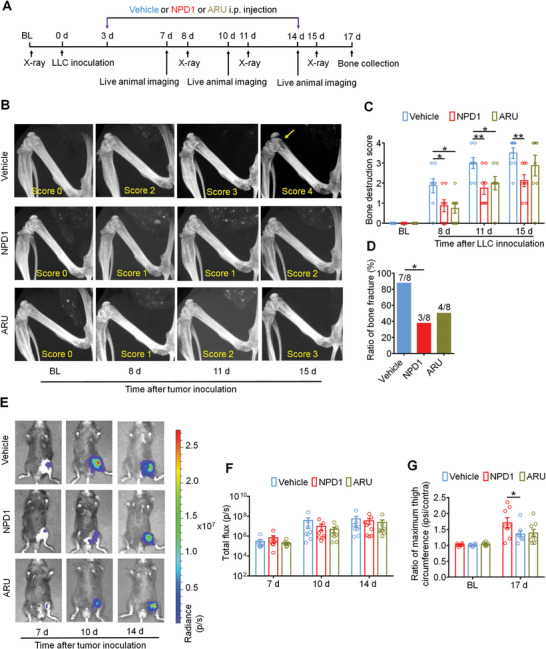
GPR37 agonists protect against cancer induced bone destruction without affecting tumor burden. A) Experimental diagram for continuous i.p. administration of vehicle, NPD1 (25 µg kg^−1^) or ARU (25 mg kg^−1^). B) Representative images showing bone destruction assessed by X‐ray. Bone destruction score is indicated in each image and arrows show bone lesions with scores over 3. C) Quantification for bone destruction scores. D) Comparison of ratio of bone fracture from tumor bearing femora taken from vehicle, NPD1 or ARU‐treated mice on day 17 after LLC inoculation. E,F) In vivo bioluminescence imaging showing no effects of vehicle, NPD1 or ARU treatment on total flux of LL/2‐Luc2 bearing femur on days 7, 10, and 14 after tumor inoculation (F). Images (E) were acquired at 15 min after i.p. injection of d‐luciferin (30 mg kg^−1^). G) Comparison of ratio of maximum thigh circumference in mice with indicated treatment on day 17 after LLC implantation. n = 8 mice/group for A‐G. Data are expressed as mean ± SEM, and analyzed using repeated‐measures two‐way ANOVA with Bonferroni's post‐hoc test (C, F, G), or two‐sided Fisher's exact test (D), *p < 0.05, **p < 0.01, ***p < 0.001.

Since bone metastasis from lung cancer leads to both bone pain and osteolytic bone lesions,^[^ ^18^ ^]^ we further assessed LLC‐induced bone destruction through continuous radiography (**Figure** **3**A). The extent of bone destruction was evaluated using X‐ray radiographs of tumor‐bearing femora, employing a scoring system ranging from 0 to 5, as outlined by Honore et al.^[^ ^19^ ^]^ Results showed that repeated i.p. administration of NPD1 and ARU significantly decreased the bone destruction score on day 8, 11, and 15 after tumor inoculation (Figure 3B,C).

Cancer‐induced osteolytic bone destruction often results in bone fractures, a significant aspect of skeletal‐related events (SREs) in patients with bone metastasis and is linked to reduced overall survival.^[^ ^20^ ^]^ On day 17 post‐LLC inoculation, mice were euthanized, and femora bearing tumors were harvested for analysis, focusing on the distal tumor‐bearing femur where bone destruction occurs. Notably, we observed that 87.5% (7/8 mice) of vehicle‐treated mice experienced bone fractures, whereas only 37.5% (3/8 mice) of those treated with NPD1 and 50% (4/8 mice) treated with ARU developed distal bone fractures (Figure 3D).

We also evaluated the development of local tumor burden after the application of vehicle, NPD1, and ARU. Results from in vivo bioluminescence imaging showed that NPD1 and ARU treatment failed to affect the total flux of LL/2 (LLC)‐Luc2 cells in tumor‐bearing femora on day 7, 10, and 14 after tumor inoculation (Figure 3E,F). Since GPR37 was reported to be expressed on macrophages, we further detected the change of tumor associated macrophages (TAMs) in tumor tissue through flowcytometry; however, no statistical differences were found in the percentage of total TAMs (CD11b^+^ F4/80^+^), M1 like TAM (CD206^−^ CD86^+^) or M2 like TAM (CD206^+^ CD86^−^) among the vehicle, NPD1 or ARU treatment (Figure S2, Supporting Information).

By day 17, tumor growth extending beyond the boundaries of the impaired distal femur became visually evident, resulting in an expansion of the circumference in the thigh affected by the tumor (ipsilateral) compared to the unaffected contralateral side. To assess this, we calculated the ratio of the maximum thigh circumference on the ipsilateral side to that on the contralateral side, providing an accurate measure of local bone destruction caused by tumor progression. Notably, our observations revealed that NPD1 treatments decreased this ratio on day 17 in LLC‐induced bone cancer models (Figure 3G). Taken together, GPR37 activation effectively protects against cancer‐induced bone destruction without significantly inhibiting local tumor growth.

### 2.4 GPR37 Agonist Therapy Protects Against Breast Cancer Induced Bone Pain and Bone Destruction

In addition to lung cancer, breast cancer can also metastasize to the bones, leading to osteolytic bone destruction and bone pain.^[^ ^21^, ^22^, ^23^ ^]^ To investigate whether GPR37 activation could offer protection against breast cancer‐induced bone destruction, we employed the medullary breast carcinoma cell line E0771 to create a syngeneic mouse model. This model was established in female C57BL/6 mice, as 98% of breast cancers occur in females. Mice were continuously i.p. injected with vehicle, NPD1 or ARU from day 3 to day 14 after E0771 intra‐femur inoculation (Figure S3A, Supporting Information). Behavioral testing showed that both NPD1 and ARU markedly attenuated mechanical allodynia and cold allodynia on day 7, 10, and 14 after tumor implantation (Figure S3B–D, Supporting Information). Meanwhile, NPD1 and ARU therapy reduced spontaneous pain compared to vehicle treatment on day 14 post‐tumor injection (Figure S3E,F, Supporting Information). X‐ray radiography of tumor‐bearing femurs showed a reduced bone destruction score in mice treated with NPD1 or ARU compared with the vehicle group on days 8 and 15 after tumor inoculation (Figure S3G, Supporting Information). Therefore, GPR37 agonists have the potential to safeguard against cancer‐induced bone pain and bone destruction triggered by various cancer subtypes susceptible to osteolytic bone metastasis.

### 2.5 Protective Effect of NPD1 and ARU is GPR37 Dependent

**Figure 4:**
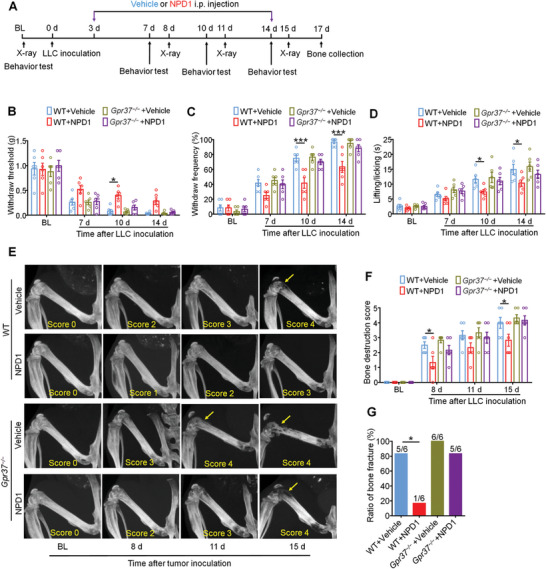
GPR37 mediates the pain relief and bone protection from NPD1 treatment. A) Study design to detect the protective effect of i.p. injection of NPD1 (25 µg kg^−1^) in WT or *Gpr37^−/−^* mice. B,C) Mechanical allodynia measured by withdrawal threshold (B) and withdrawal frequency (C) on day 7, 10, and 14 after tumor inoculation. D) Cold allodynia via acetone response testing. E,F) Representative X‐ray images (E) and quantification of bone destruction score (F) in WT or *Gpr37^−/−^* mice applied with vehicle or NPD1 (25 µg kg^−1^) on day 8, 11 and 15 post LLC inoculation. Bone destruction score is indicated in each image and arrows show bone lesions with scores over 3. G) Comparison of ratio of bone fracture in tumor bearing femora on day 17 after LLC implantation. n = 6 mice/group. All data displayed represent the mean ± SEM, and are analyzed using repeated‐measures two‐way ANOVA with Bonferroni's post‐hoc test (B, C, D, F), or two‐sided Fisher's exact test (G), *p < 0.05, ***p < 0.001.

To verify whether the antinociceptive and bone‐protective effects of NPD1 are GPR37 dependent, wild‐type (WT) mice and GPR37 knockout (*Gpr37* ^−/−^) mice were inoculated with LLC cells intrafemorally followed by continuous i.p. injection of vehicle or NPD1 from day 3 to day 14 post‐tumor inoculation (**Figure** **4**A; Figure S4A, Supporting Information). Notably, WT mice treated with NPD1 or ARU displayed an increased hindpaw withdrawal threshold and decreased withdrawal frequency compared to vehicle treatment. However, the attenuated mechanical allodynia caused by NPD1 and ARU was abolished in *Gpr37* ^−/−^ mice (Figure 4B,C; Figure S4B,C, Supporting Information). Similarly, NPD1 or ARU treatment alleviated cold allodynia in the acetone response test in WT mice but not in *Gpr37* ^−/−^ mice (Figure 4D; Figure S4D, Supporting Information). Regarding cancer‐induced bone destruction, X‐ray radiography showed that NPD1 or ARU significantly improved the lesions of cancer‐bearing femora in WT mice on day 8, 15 after LLC injection; however, in *Gpr37* ^−/−^ mice, NPD1 and ARU failed to protect against bone destruction compared to vehicle treatment (Figure 4E,F; Figure S4E,F, Supporting Information). Moreover, application of NPD1 or ARU reduced the ratio of bone fracture in WT mice but not in *Gpr37* ^−/−^ mice on day 17 post‐tumor implantation (Figure 4G; Figure S4G, Supporting Information). To be noted, there is no difference in the locomotor function between WT mice and *Gpr37* ^−/−^ mice at baseline and on day 14 after tumor inoculation, indicate GPR37 pathway does not affect the locomotor function directly (Figure S5A,B, Supporting Information).

We also evaluated the role of GPR37 in the instant analgesic effect of NPD1 and ARU when i.t. injected into WT mice or *Gpr37* ^−/−^ mice on day 11 after LLC inoculation. NPD1 treatment significantly reduced mechanical allodynia in WT mice at 1 h, 3 h, and 5 h after i.t. injection. However, the analgesic effect of NPD1 was abolished in *Gpr37* ^−/−^ mice (Figure S6A,B, Supporting Information). Additionally, reduced mechanical allodynia was detected in WT mice but not in *Gpr37* ^−/−^ mice after a single dose of ARU i.t. injection (Figure S6C,D, Supporting Information). These findings illustrate that the protective effect of NPD1 and ARU in metastatic bone cancer is GPR37 dependent.

### 2.6 Increased Expression of GPR37 in DRG Neurons in BCP Model

**Figure 5:**
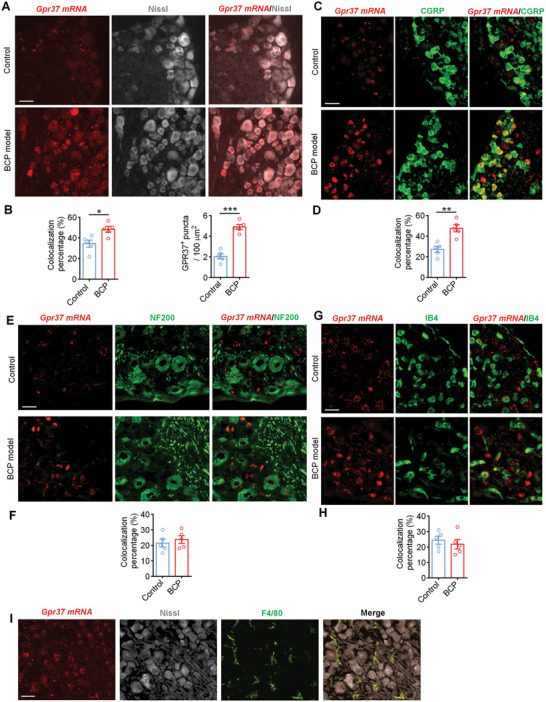
Increased expression of GPR37 in DRG neurons from tumor bearing mice. A) Representative in situ hybridization images showing GPR37 mRNA expression in L3‐L5 DRG neurons (Labelled by Nissl) from control mice or mice with BCP. Scale bar, 40 µm. B) Quantification for panel (A). Left, the percentage of *Gpr37* mRNA positive neurons to Nissl labelled cells. Right, the average No. of *Gpr37* mRNA positive puncta per 100 µm^2^ in the counted field. C) Colocalization of *Gpr37* mRNA with CGRP in DRG neurons from naïve mice or mice with BCP. Scale bar, 40 µm. D) Quantification of for panel (C) showing the percentage of *Gpr37* mRNA positive neurons to CGRP^+^ DRG neurons. E) Co‐expression of *Gpr37* mRNA with NF200 in DRG neurons from naïve mice or BCP mice. Scale bar, 40 µm. F) Quantification of for panel (E). G‐H) Representative images (G) and quantification (H) exhibiting the colocalization of *Gpr37* mRNA and IB4 in DRG neurons from naïve or BCP mice. Scale bar, 40 µm. I) Representative images showing the co‐expression of *Gpr37* mRNA, Nissl and F4/80 in DRG neurons from tumor bearing mice. Scale bar, 40 µm. n = 4 mice/group for A‐H. Data displayed represent the mean ± SEM, and are analyzed with two‐tailed Student's *t*‐test, *p < 0.05, **p < 0.01, ***p < 0.001.

GPR37 has been reported to be expressed in several cell types of the neuronal system, including oligodendrocytes, astrocytes, macrophages, and neurons.^[^ ^9^ ^]^ Here, we examined the expression of GPR37 in the dorsal root ganglion (DRG) from mice with BCP. On day 11 after tumor inoculation, the mice were sacrificed, and DRG neurons were collected for in situ hybridization (RNAscope) and immunostaining. Quantitative analysis demonstrated that compared with naïve control mice, there was a significant increase in the expression of GPR37 detected via in situ hybridization in DRG neurons (labeled with Nissl) from mice with BCP (**Figure** **5**A,B). Especially, GPR37 is mainly increasingly expressed in neurons labeled with calcitonin gene‐related peptide (CGRP), a marker of peptidergic nociceptors after the development of BCP (Figure 5C,D). Further detection revealed the colocalization of GPR37 with neurofilament 200 (NF200) which labels large diameter Aβ sensory neurons and IB4 which labels non‐peptidergic neurons; however, the colocalization percentages showed no differences between naïve mice and BCP mice (Figure 5E‐H).

We also detected the expression of GPR37 via immunostaining, RT‐PCR and western blotting, and verified its elevated expression in BCP model (Figure S7A–D, Supporting Information). The co‐expression of GPR37 with CGRP, NF200 and IB4 in DRG neurons from mice with BCP were also confirmed via IF (Figure S7E, Supporting Information). The size distribution of DRG neurons revealed that GPR37 is mainly expressed in small‐diameter neurons (Figure S7F, Supporting Information). In *Gpr37* ^−/−^ mice with LLC injection, the expression of GPR37 is absent in DRG neurons via in situ hybridization, IF staining, western blotting and PCR (Figure S7G–J, Supporting Information). The colocalization of GRP37 with F4/80 and Nissl in DRG was also detected via RNAscope (Figure 5I), which indicated that GPR37 is expressed in both neurons and macrophages in DRG from mice with BCP.

### 2.7 NPD1 and ARU Reduce Hyperexcitability of DRG Neurons from BCP Mice via GPR37

**Figure 6:**
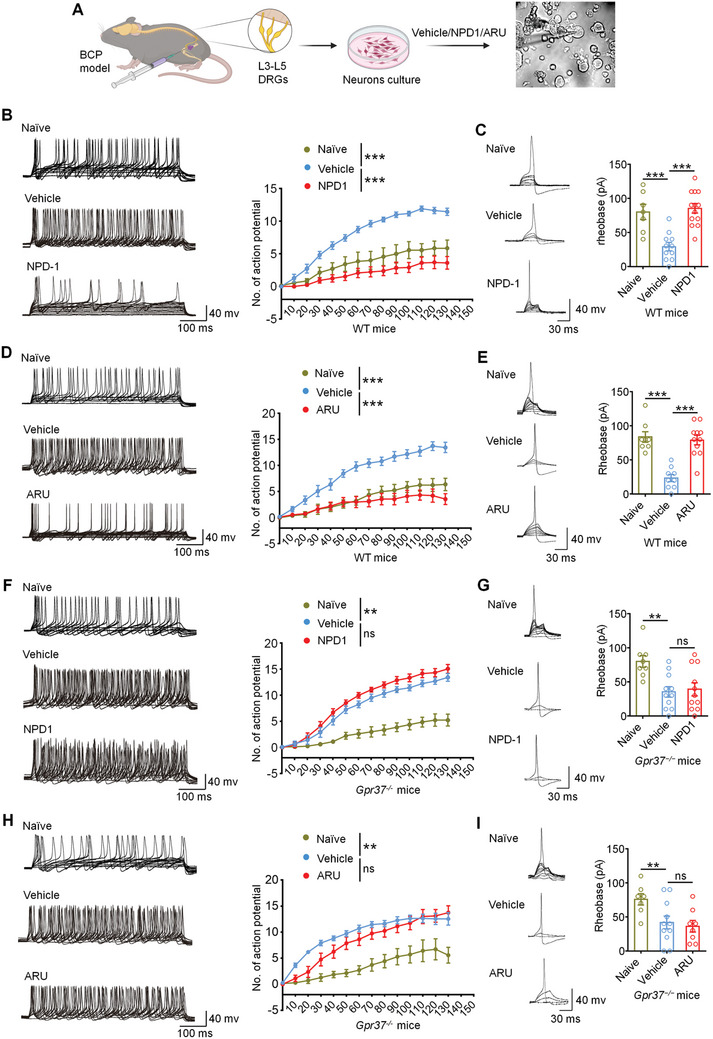
NPD1 and ARU impaired the activity of small‐sized DRG neurons in BCP mice. A) Schematic of DRG neurons preparation, drug treatment, and bright field image with a recording micropipette sealed on a small‐diameter neuron (nociceptor). B) Representative current‐evoked action potential (AP) traces (left) and quantification of the firing rate (right) in a small DRG neuron from WT naïve mice, BCP mice after vehicle or NPD1 perfusion (10 ng mL^−1^, 2 min). C) Representative traces of rheobases (left) and comparison of the averages of rheobases (right) from naïve mice, vehicle or NPD1 treated group (naïve: n = 7 neurons/5 mice; vehicle: n = 9 neurons/5 mice; NPD1: n = 13 neurons/5 mice). D) Representative current‐evoked AP traces (left) and quantification of the No. of AP (right) in small DRG neurons from WT naïve mice, BCP mice after vehicle or ARU perfusion (10 µM, 2 min). E) Representative traces of rheobases (left) and comparison of the averages of rheobases (right) from naïve mice, vehicle or ARU treated group (naïve: n = 8 neurons/5 mice; vehicle: n = 9 neurons/5 mice; ARU: n = 11 neurons/5 mice). F) Representative current‐evoked AP traces (left) and comparison of the firing rate (fight) in small‐diameter DRG neuron from *Gpr37^−/−^* naïve mice, *Gpr37^−/−^* BCP mice after vehicle or NPD1 perfusion. G) Representative traces of rheobases (left) and comparison of the averages of rheobases (right) from *Gpr37^−/−^* naïve mice, vehicle or NPD1 treated group (naïve: n = 8 neurons/5 mice; vehicle: n = 11 neurons/5 mice; NPD1: n = 11 neurons/5 mice). H) Representative current‐evoked AP traces (left) and comparison of the firing rate (right) in small‐diameter DRG neuron from *Gpr37^−/−^* naïve mice, *Gpr37^−/−^* BCP mice after vehicle or ARU perfusion. I) Representative traces of rheobases (left) and comparison of the averages of rheobases (right) from *Gpr37^−/−^* naïve mice, vehicle or ARU treated group (naïve: n = 7 neurons/5 mice; vehicle: n = 11 neurons/5 mice; ARU: n = 8 neurons/5 mice). Data are presented as mean ± SEM, and analyzed using repeated‐measures two‐way ANOVA with Bonferroni's post‐hoc test (B, D, F, H), or one‐way ANOVA with Bonferroni's post‐hoc test (C, E, G, I); ns, not significant; **p < 0.01, ***p < 0.001.

Since cancer‐induced bone pain is transduced by peripheral nociceptors in the DRG, we sought to determine whether GPR37 signaling in sensory neurons contributes to the antinociceptive effects of NPD1 and ARU in BCP. Given that the application of NPD1 and ARU directly alleviates acute bone pain via i.t. injection, we hypothesized that GPR37 activation may acutely attenuate bone cancer‐induced hyperexcitability of peripheral nociceptors. To test this hypothesis, WT mice and *Gpr37* ^−/−^ mice were implanted with LLC cells to establish BCP models, and lumbar L3–L5 DRGs were isolated on day 11 for neuron culture. DRG neurons were further incubated with vehicle, NPD1, or ARU for 2 h followed by patch clamp recordings (**Figure** **6**A). Upon examination of current evoked action potentials, we found that while vehicle‐treated DRG neurons from tumor‐bearing mice exhibited increased No. of action potentials relative to naïve (non‐tumor‐bearing) mice, NPD1 or ARU could completely reverse this cancer‐induced nociceptor hyperexcitability (Figure 6B,D). In addition, both NPD1 and ARU incubation exhibited increased rheobase of action potential compared with vehicle treatment in DRG neurons from WT mice with BCP (Figure 6C,E).

Importantly, in DRG from *Gpr37* ^−/−^ mice with bone cancer, the nociceptors remain their hyperexcitable state compared to DRG neurons from naïve mice; however, neither NPD1 nor ARU affected the firing rate or rheobase when compared to vehicle incubation (Figure 6F–I). These findings indicate that NPD1 and ARU directly reduce the excitability of DRG neurons from cancer‐bearing mice in a GPR37‐dependent manner.

### 2.8 GPR37 Modulates Cancer Induced Osteoclast Differentiation through IL‐10

**Figure 7:**
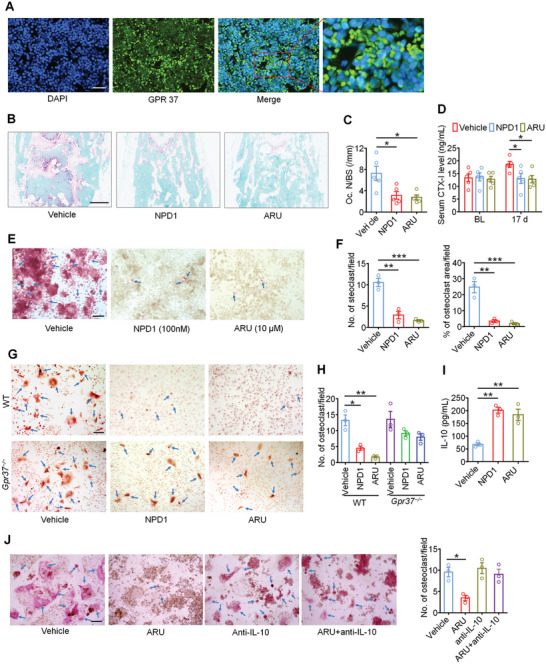
GPR37 agonist inhibit osteoclastogenesis through IL‐10. A) Immunostaining showed wide expression of GPR37 in bone marrow cells from murine femur section. Scale bar, 50 µm. B,C) Representative images (B) and quantification (C) of TRAP staining to reveal osteoclast numbers in the tumor‐bearing distal femora from mice continuously treated with vehicle, NPD1 (25 µg kg^−1^) or ARU (25 mg kg^−1^) measured on day 11 after LLC inoculation. n = 5 mice/group. Scale bar, 500 µm. D) Level of serum CTX‐I at baseline or on day 17 after tumor implantation. n = 5 mice/group. E,F) TRAP staining revealing osteoclast numbers after differentiation from RAW264.7 cells stimulated with 35 ng mL^−1^ RANKL, in the presence of vehicle, NPD1 (100 nM) or ARU (10 µM). Arrows indicate TRAP^+^ multinucleated osteoclasts. E Representative TRAP‐stained images. F quantification (n = 3 biologically independent experimental replicates). Scale bar, 200 µm. G,H) TRAP staining for osteoclasts differentiated from BMDM cells from WT mice or *Gpr37^−/−^* mice, each treated with vehicle, NPD1 (100 nM) or ARU (10 µM). RANKL: 35 ng mL^−1^, MCSF: 20 ng mL^−1^. G Representative images of TRAP staining. Arrows indicate TRAP^+^ osteoclasts. Scale bar, 100 µm. H Quantification for (G), n = 3 independent cultures. I) Measurement of IL‐10 level in the culture medium of Raw 264.7 cells 24 h after vehicle, NPD1 (100 nM) or ARU (10 µM) co‐incubation with RANKL (35 ng mL^−1^). n = 3 biologically independent experimental replicates. J) TRAP staining showing No. of osteoclasts differentiated form Raw 264.7 cells treated with vehicle, ARU (10 µM), anti‐IL‐10 antibody (1 µg mL^−1^) or ARU + anti‐IL‐10 antibody. Left, representative images and arrows indicate TRAP^+^ osteoclasts.; right, quantification. Scale bar, 200 µm. n = 3 biologically independent experimental replicates. Data indicate the mean ± SEM, and are analyzed using one‐way ANOVA with Bonferroni's post‐hoc test (C, F, H, I, J), or repeated‐measures two‐way ANOVA with Bonferroni's post‐hoc test (D), *p < 0.05, **p < 0.01, ***p < 0.001.

Cancer‐induced osteoclast activation could promote osteolytic bone destruction and bone pain.^[^ ^24^ ^]^ We then tested the potential effect of NPD1 and ARU on osteoclast differentiation. First, through immunostaining, we found that GPR37 is highly expressed in murine monocyte/macrophage cell line – RAW 264.7 cells and bone marrow‐derived macrophages (BMDM), which are typically preosteoclasts (Figure S8A,B, Supporting Information). Moreover, GPR37 expression was also detected in the bone marrow tissue from femora of WT mice (**Figure** **7**A). On day 11 after LLC inoculation, tumor‐bearing femora were harvested for tartrate‐resistant acid phosphatase (TRAP) staining to assess cancer‐induced osteoclast activation. Results indicated that both NPD1 and ARU treatment could significantly inhibit the number of osteoclasts in the distal tumor‐bearing femora compared to vehicle treatment (Figure 7B,C). Serum levels of CTX‐I, which is a marker of bone resorption, also decreased after the application of NPD1 and ARU on day 17 after LLC inoculation (Figure 7D). These findings imply that NPD1 and ARU could inhibit osteolytic bone destruction via directly suppressing cancer‐induced osteoclastogenesis.

Given that systemic NPD1 and ARU treatment reduces osteoclast numbers in vivo, we sought to determine the role of GPR37 activation in osteoclast differentiation in vitro. Murine macrophage RAW 264.7 cells were treated with RANKL (35 ng mL^−1^, for 6 days) to promote osteoclastogenesis. Importantly, the presence of NPD1 (100 nM) or ARU (10 µM) significantly inhibited osteoclast formation compared with vehicle treatment (Figure 7E,F). We also harvested and cultured BMDMs from WT or *Gpr37* ^−/−^ mice, which were further induced into osteoclasts with 20 ng mL^−1^ MCSF and 35 ng mL^−1^ RANKL for 7 days. TRAP staining showed that NPD1 and ARU treatment significantly inhibited osteoclast differentiation from BMDM from WT mice but not from *Gpr37* ^−/−^ mice (Figure 7G,H). These findings indicated that NPD1 and ARU inhibit osteoclastogenesis via the GPR37 pathway.

It was reported that NPD1, when binding to GPR37 in macrophages, promotes the release of IL‐10, which is identified as an anti‐osteoclastogenesis cytokine.^[^ ^10^, ^25^ ^]^ We hereby detected the secretion of IL‐10 in the culture system of RAW 264.7 cells induced with RANKL. Notably, incubation with NPD1 and ARU significantly increased the level of IL‐10 in the culture medium (Figure 7I). To verify the role of IL‐10 in GPR37‐mediated blocking of osteoclast differentiation, we added anti‐IL‐10 neutralizing antibodies into the induction medium of RAW 264.7 cells. Results showed that the anti‐IL‐10 antibody could reverse the inhibition of osteoclast formation by ARU (Figure 7J). Similarly, in osteoclast induction systems using BMDMs from WT mice, but not *Gpr37* ^−/−^ mice, the suppression of osteoclast differentiation by NPD1 was also reversed by the addition of anti‐IL‐10 antibodies (Figure S8C,D, Supporting Information). Taken together, these data indicate that GPR37 activation in macrophages suppresses osteoclastogenesis through the production of IL‐10.

### 2.9 β‐Arrestin 2 Mediates IL‐10 Production and Neuronal Modulation Downstream of GPR37 Activation

**Figure 8:**
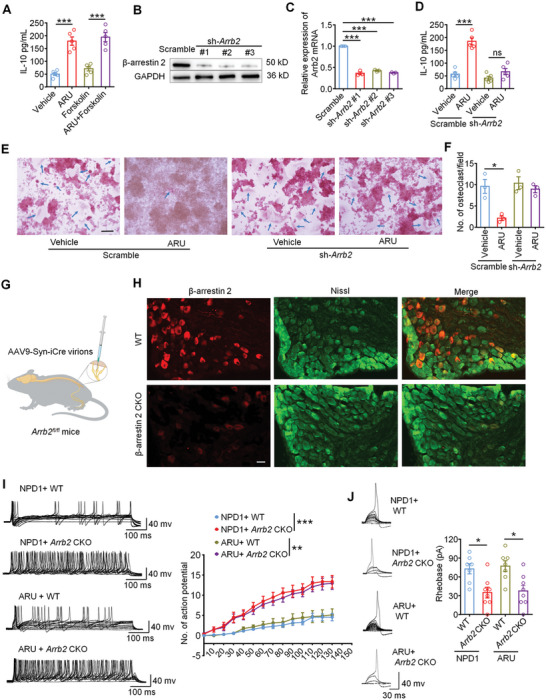
β‐arrestin 2 mediates IL‐10 production and neuronal modulation of GPR37 activation. A) IL‐10 level in the culture medium of Raw 264.7 cells 24 h after vehicle, ARU (10 µM), Forskolin (1 µM) or ARU + Forskolin co‐incubation with RANKL (35 ng mL^−1^). n = 5 biologically independent experimental replicates. B) Western blot showing decreased expression of β‐arrestin 2 in shRNAs treated Raw 264.7 cells. C) RT‐PCR determining reduced level of β‐arrestin 2 mRNA in shRNAs treated Raw 264.7 cells. n = 3 independent experimental replicates. D) Measurement of IL‐10 level in the culture medium of scramble or *Arrb2*‐shRNA‐treated RAW 264.7 cells administered with Vehicle or ARU (10 µM) and with RANKL (35 ng mL^−1^). n = 5 biologically independent experimental replicates. E‐F) TRAP staining for osteoclasts differentiated from scramble or *Arrb2*‐shRNA‐treated RAW 264.7 cells, each treated with vehicle or ARU (10 µM). RANKL: 35 ng mL^−1^. E Representative images of TRAP staining. Arrows indicate TRAP^+^ osteoclasts. Scale bar, 200 µm. F Quantification for (E), n = 3 biologically independent experimental replicates. G) Schematic for the establishment of *Arrb2* conditional knockout (CKO) mice via DRG injection. H) IF staining showing β‐arrestin 2 expression in DRG neurons from naïve mice or β‐arrestin 2 CKO mice with BCP. Scale Bar, 50 µm. I) Representative current‐evoked AP traces (left) and quantification of the firing rate (right) in small‐diameter DRG neurons from WT mice or β‐arrestin 2 CKO BCP mice after NPD1 (10 ng mL^−1^, 2 min) or ARU perfusion (10 µM, 2 min). J) Representative traces of rheobases (left) and comparison of the averages of rheobases (right) from WT mice or β‐arrestin 2 CKO BCP mice after NPD1 or ARU perfusion. (n = 7–8 neurons/5 mice, each group). Data are expressed as mean ± SEM, and analyzed using one‐way ANOVA with Bonferroni's post‐hoc test (A, C, D, F, J), or repeated‐measures two‐way ANOVA with Bonferroni's post‐hoc test (I), *p < 0.05, **p < 0.01, ***p < 0.001.

Next, we try to exploring the downstream pathways of GPR37 activation, particularly its modulation of IL‐10 release. GPR37, as a Gi/o‐coupled receptor, typically inhibits adenylyl cyclase (AC) upon activation, thereby reducing intracellular cAMP levels.^[^ ^8^, ^26^ ^]^ Based on this, we first examined whether AC plays a role in IL‐10 production following GPR37 activation. Using an osteoclast induction system with RAW 264.7 cells, we treated the cells with the AC agonist Forskolin (1 µM) in the presence or absence of ARU. The results demonstrated that Forskolin failed to reverse ARU‐induced IL‐10 release (**Figure** **8**A), indicating that AC inhibition is not involved in GPR37‐mediated IL‐10 production.

It is well‐established that upon activation, Gi/o‐coupled GPCRs undergo phosphorylation by GPCR kinases (GRKs), creating binding sites for β‐arrestins.^[^ ^8^, ^27^ ^]^ β‐arrestins, particularly β‐arrestin 2, function as multifunctional adaptor proteins that mediate receptor desensitization, internalization, and scaffolding for alternative signaling cascades, such as ERKs, JNKs, and MAPKs.^[^ ^28^, ^29^ ^]^ To further evaluate the role of β‐arrestin 2 in GPR37‐mediated IL‐10 production, we knocked down β‐arrestin 2 expression in RAW 264.7 cells using shRNA (Figure 8B,C). ARU was then administered to scramble or Arrb2‐shRNA‐treated RAW 264.7 cells during osteoclast induction. Our results showed that the ARU‐induced increase in IL‐10 was significantly reduced in β‐arrestin 2 knockdown cells (Figure 8D). In addition, in vitro TRAP staining confirmed that ARU inhibited osteoclast differentiation in scramble‐treated cells but not in β‐arrestin 2 knockdown cells (Figure 8E,F). These findings demonstrate that β‐arrestin 2 recruitment is critical for GPR37‐mediated IL‐10 production and osteoclastogenesis inhibition.

To further validate this mechanism, we analyzed the tumor single‐cell RNA sequencing data (GSE202051, Figure S9A, Supporting Information). t‐SNE plots revealed that GPR37 was specifically expressed in macrophages, particularly within the c_0 subcluster (Figure S9B–D, Supporting Information). Differential expression and enrichment analyses between GPR37‐positive and GPR37‐negative macrophages identified significant enrichment of β‐arrestin pathway‐related genes, especially ARRB1 and ARRB2, within the c_0 subcluster (Figure S9E, Supporting Information). Furthermore, the expression patterns of GPR37 and IL10 were consistent with the β‐arrestin pathway enrichment scores (Figure S9F–H, Supporting Information), reinforcing the involvement of β‐arrestin in GPR37‐mediated IL‐10 regulation.

Additionally, we explored whether the effects of GPR37 activation on nociceptors are β‐arrestin 2 dependent. *Arrb2* ^fl/fl^ mice were injected with AAV‐hSyn‐cre virions into the lumbar DRGs to conditionally knockout (CKO) β‐arrestin 2 in L3‐L5 DRGs (Figure 8G). BCP was then induced by femoral inoculation of LLCs, and L3‐L5 DRG neurons were harvested for culture 14 days after tumor injection. Immunofluorescence staining confirmed efficient β‐arrestin 2 knockout in DRGs from *Arrb2* CKO mice (Figure 8H). Patch clamp recordings revealed that neurons from *Arrb2* CKO mice displayed a significantly increased No. of action potentials compared to WT neurons when treated with NPD1 or ARU (Figure 8I). Moreover, the rheobase of action potentials in *Arrb2* CKO neurons was reduced compared to WT neurons under the same conditions (Figure 8J). In summary, these findings collectively indicate that GPR37 activation alleviates neuronal hyperexcitability and promotes IL‐10 production through a β‐arrestin 2‐dependent mechanism, providing novel insights into the molecular regulation of bone cancer pain.

### 2.10 NPD1 and ARU Suppress Synaptic Nociceptive Transmission in the Spinal Cord via GPR37

**Figure 9:**
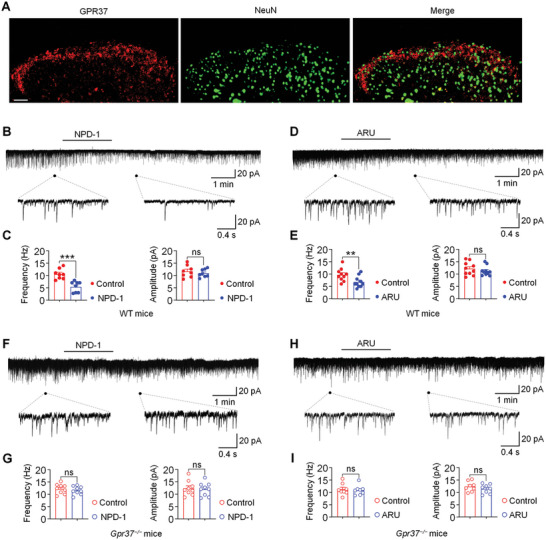
Effect of NPD1 and ARU on sEPSCs in spinal dorsal horn from BCP mice. A) Immunostaining showed the expression of GPR37 and NeuN in lamina I‐II of the spinal dorsal horn (SDH) from mice with BCP. Scale bar, 50 µm. B) Chart recording showing spontaneous excitatory postsynaptic currents (sEPSCs) in the absence and presence of NPD1 (10 ng mL^−1^, 2 min) obtained from WT BCP mouse. C) The average of sEPSCs frequency (left) and amplitude (right) before (control) and 4–5 min after the beginning of NPD1 perfusion, respectively. D) Chart recording showing sEPSCs in the absence and presence of ARU (10 µM, 2 min) obtained from WT BCP mouse. E) The average of sEPSCs frequency (left) and amplitude (right) before (control) and 4–5 min after the beginning of ARU perfusion, respectively. F) Chart recording showing sEPSCs in the absence and presence of NPD1 (10 ng mL^−1^, 2 min) obtained from *Gpr37^−/−^* BCP mouse. G) The average of sEPSCs frequency (left) and amplitude (right) before (control) and 4–5 min after the beginning of NPD‐1 perfusion, respectively. H) Chart recording showing sEPSCs in the absence and presence of ARU (10 µM, 2 min) obtained from *Gpr37^−/−^* BCP mouse. I) The average of sEPSCs frequency (left) and amplitude (right) before (control) and 4–5 min after the beginning of ARU perfusion, respectively. The duration of drug perfusion is indicated by a horizontal bar above the chart recording, while consecutive traces of spontaneous events are shown at an expanded time scale, marked by a short bar below the recording. n = 8–10 neurons from 6 mice for B‐I. Data are presented as mean ± SEM, and analyzed using two‐tailed Student's t‐test; ns, not significant; **p < 0.01, ***p < 0.001.

We also examined the expression and role of GPR37 in the spinal dorsal horn (SDH) from mice with BCP. Immunostaining showed that GPR37 is highly expressed in the sensory nerve terminals that project to lamina I‐II in the SDH (**Figure** **9**A). Patch clamp recordings showed that perfusion of spinal cord slices with NPD1 or ARU significantly decreased the frequency of spontaneous excitatory postsynaptic currents (sEPSCs) from lamina II interneurons. There was no change in the amplitude of sEPSCs, which implies that the effect of NPD1 and ARU mainly relies on presynaptic regulation (Figure 9B‐E). In sharp contrast, superfusion of NPD1 or ARU to spinal cord slices from *Gpr37* ^−/−^ mice inoculated with LLC failed to affect the frequency and amplitude of sEPSCs from lamina II interneurons (Figure 9F–I). Thus, NPD1 and ARU also have an active role in modulating spinal nociceptive transmission dependent on the GPR37 pathway.

### 2.11 Endogenous NPD1 is Negatively Correlated with Bone Pain and Serum CTX‐I Level in Cancer Patients with Bone Metastasis

**Figure 10:**
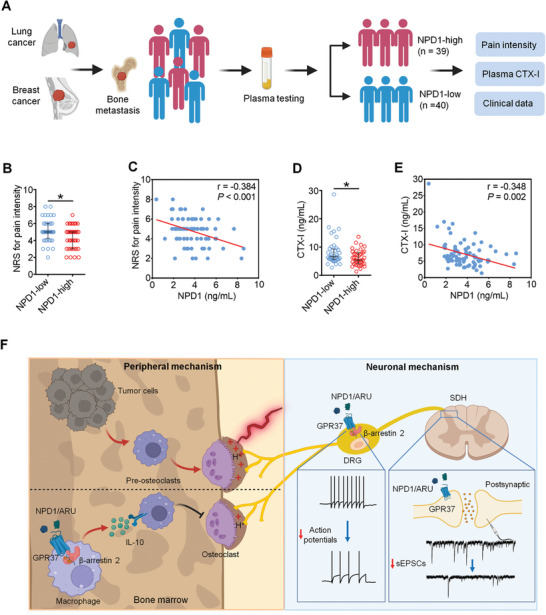
NPD1 is negatively correlated with cancer pain and CTX‐I level in patients with metastatic cancer. A) Schematic diagram for the design of the clinical study. B) Comparison of pain intensity via number rating score (NRS) in cancer patients with bone metastasis. These patients were divided into NPD1‐low and NPD1‐high groups according to their levels of serum NPD1. n = 40 patients in NPD1‐low group and n = 39 patients in NPD1‐high group. C) Correlation between serum NPD1 level and NRS score in the enrolled patients. n = 79 patients. D) Elisa measurement of plasma CTX‐I level in NPD1‐low (n = 40) and NPD1‐high (n = 39) patients. E) Correlation between plasma NPD1 level and plasma CTX‐I level in the enrolled patients (n = 79). F) Graphical abstract of the current study. GPR37 activation reduces cancer pain and cancer induced bone destruction via peripheral and neuronal mechanism. NPD1 or ARU acting on GPR37 expressed in macrophage could promote the release of IL‐10 via recruitment of β‐arrestin 2 which further inhibit cancer induced ostoclastogenesis. Moreover, GPR37 activation in DRG could suppress No. of action potentials via β‐arrestin 2 pathway, while GPR37 activation in SDH decreases the frequency of sEPSCs, both leading to the inhibition of cancer induced neuronal hyperexcitability. Data in A and C are expressed as median (interquartile range), and analyzed with Mann‐Whitney test (B, D) and Person correlation test (C, E), *p < 0.05.

Since NPD1 could be synthesized and released under both physical and pathological conditions,^[^ ^30^ ^]^ we measured endogenous NPD1 levels in serum and cerebrospinal fluid (CSF) from mice before and after BCP establishment. The results showed a decrease in serum NPD1 levels following BCP development, while NPD1 levels in CSF remained unchanged (Figure S10A,B, Supporting Information). We further investigated the correlation between endogenous NPD1 levels and pain intensity in patients with bone metastasis from lung cancer or breast cancer (**Figure** **10**A). Blood samples were obtained to detect the levels of serum NPD1 and CTX‐I. The assessment of pain intensity was conducted using the Numerical Rating Scale (NRS) score.^[^ ^31^ ^]^ According to their plasma NPD1 levels (median 3.62 ng mL^−1^), patients were further allocated into NPD1‐low (≤3.62 ng mL^−1^) and NPD1‐high (>3.62 ng mL^−1^) groups. There was no statistical difference in general information such as age, sex, Body Mass Index (BMI), type of primary cancer, number of bone lesions, and ratio of surgical treatment between the two groups (Table S1, Supporting Information). However, patients with high levels of NPD1 experienced less pain compared to NPD1‐low patients (Figure 10B). A negative correlation was observed between serum NPD1 levels and NRS scores for all participants (Figure 10C). Moreover, the serum CTX‐I levels in NPD1‐high patients were significantly lower than those in NPD1‐low patients (Figure 10D). Further analysis showed that the serum concentration of ND1 was negatively correlated with the level of CTX‐I in the enrolled patients (Figure 10E). These results from cancer patients further verify the protective effect of NPD1 in cancer induced bone pain.

## 3 Discussion

Osteolytic BCP presents in various forms, including acute breakthrough pain and persistent background pain. The mechanism of BCP is complex, mainly involving the peripheral overactivation of osteoclasts in the tumor microenvironment (TME) and neuronal hyperexcitability in response to afferent noxious signals in the DRG and SDH.^[^ ^32^, ^33^ ^]^ In the present study, we found that activation of GPR37 by NPD1 or ARU could alleviate both acute and persistent BCP. GPR37 activation could also protect against cancer‐induced bone destruction. Mechanistically, NPD1 or ARU acting on GPR37 expressed in macrophages could promote the release of IL‐10 via recruitment of β‐arrestin 2, which further inhibits cancer‐induced osteoclastogenesis. Meanwhile, direct GPR37 activation on DRG neurons and afferent terminals in the SDH suppresses the firing of action potentials and the frequency of sEPSCs in a β‐arrestin 2 dependent manner, leading to the inhibition of cancer‐induced neuronal hyperexcitability (Figure 10F). These findings illustrate that GPR37 is a common therapeutic target combining both peripheral and central mechanisms of BCP.

GPR37 serves as a substrate for Parkin, and in Parkinson's disease patients, insoluble aggregates of GPR37 accumulate extensively in the Lewy bodies of the brain.^[^ ^34^, ^35^ ^]^ Moreover, GPR37 binds to the dopamine transporter to modulate dopamine uptake, and mutations in the GPR37 gene contribute to autism spectrum disorders.^[^ ^36^, ^37^ ^]^ Additionally, GPR37 is expressed by astrocytes in the brain and spinal cord, regulating the differentiation of these cells.^[^ ^38^ ^]^ The present study detected increased expression of GPR37 in DRG neurons, especially CGRP^+^ nociceptors in BCP mice. Activation of GPR37 by NPD1 and ARU instantly reduces the number of action potentials in DRG neurons and sEPSCs in SDH neurons. Through the inhibition of neuronal hyperexcitability, GPR37 agonists exhibit an instant analgesic effect with promising applications for the breakthrough pain of bone metastasis.

Expression and function of GPR37 on macrophage has also been increasingly investigated. Depletion of GPR37 in peritoneal macrophages triggers a shift in phenotype, transitioning from M2‐like to M1‐like macrophages.^[^ ^9^, ^39^ ^]^ These Gpr37‐deficient macrophages exhibit heightened production of pro‐inflammatory cytokines (such as IL‐6, IL‐1β, and TNF‐α) while showing diminished levels of anti‐inflammatory cytokines (including IL‐10 and TGF‐β1) in response to inflammatory stimuli. Conversely, treatment with NPD1 leads to elevated levels of IL‐10 and TGF‐β1 but reduced levels of IL‐1β, TNF‐α, and IL‐6.^[^ ^40^ ^]^ Notably, the adoptive transfer of GPR37‐activated macrophages via ARU or NPD1 significantly mitigates sepsis and malaria infections.^[^ ^14^ ^]^ Here, we further revealed the role of GPR37 in macrophages and bone marrow cells as preosteoclasts in the modulation of osteoclast differentiation. IL‐10 released from GPR37 activation successfully inhibits the overactivation of osteoclasts in the TME and thus alleviates cancer‐induced bone destruction. Moreover, we identified β‐arrestin 2 as a crucial downstream mediator which facilitates IL‐10 release and inhibits osteoclastogenesis. These findings highlight β‐arrestin 2′s role in regulating immune responses and nociception, and complete the signaling cascade of GPR37 signaling in regulation of cancer‐related bone pain.

NPD1, a derivative of Omega‐3 polyunsaturated fatty acids DHA and EPA, is synthesized and secreted physiologically.^[^ ^41^ ^]^ As member of specialized pro‐resolution mediators (SPM) family, NPD1 exhibits rapid synthesis in response to oxidative stress or ischemic brain injury, exerting neuroprotective effects through anti‐inflammation and reducing mitochondria‐mediated apoptosis. Decreased endogenous NPD1 production in diabetic wound is associated with impaired healing.^[^ ^40^ ^]^ Treatment with exogenous NPD1 provides high‐grade neurobehavioral recovery and decreases ischemic injury.^[^ ^42^, ^43^ ^]^ NPD1 was also found to alleviate neuropathic pain following nerve injury.^[^ ^15^ ^]^ In the present study, NPD1 administration alleviated both bone pain and bone destruction. Importantly, in cancer patients with bone metastasis, the plasma endogenous NPD1 level is negatively correlated with pain intensity and marker reflects bone resorption. This further verifies the bone protection effect of NPD1, and plasma NPD1 level could be a potential marker to evaluate the degree of bone destruction in patients with metastatic cancer.

It's worth noting that GPR37 is also reported to be expressed in certain cancer cells and plays a role in either the progression or regression of tumors depending on the type of tumor.^[^ ^44^, ^45^ ^]^ In our current study, GPR37 agonists did not impact the local tumor burden. However, further investigation is needed to delineate the individual and combined roles of GPR37 activation in tumor immune surveillance and tumor growth across various cancer models.

In summary, our study reveals that GPR37 activation via NPD1 or ARU alleviates bone cancer pain and improves cancer‐induced bone destruction through direct and synergistic actions on nociceptors and osteoclasts. Thus, GPR37 is a promising novel target for the clinical treatment of cancer pain in patients with bone metastasis.

## 4 Experimental Section

### Cell Culture

Murine Lewis lung carcinoma cell line with luciferase LL/2 (LLC1)‐Luc2 (ATCC CRL‐1642‐LUC2) and murine monocyte/macrophage cell line RAW 264.7 (ATCC TIB‐71) were purchased from ATCC. The mouse E0771 breast cancer cell line (94A001) was obtained from CH3 BioSystems. Cells were cultured in high glucose (4.5 g L^−1^) Dulbecco's modified Eagle medium (Gibco, Thermo Fisher Scientific), supplemented with 10% fetal bovine serum (Gibco, Thermo Fisher Scientific) and 1% antibiotic‐antimycotic solution (Sigma‐Aldrich). They were then cultured in the incubator with 5% CO_2_ at 37 °C. Blasticidin (2 µg mL^−1^, Gibco, Thermo Fisher Scientific) was added into LL/2‐Luc2 culture medium and removed 3 days before the implantation of mice.

To construct a stable *Arrb2* gene knockdown cell line in RAW 264.7 cells, we ordered three unique shRNA sequences targeting Arrb2 (5′‐3′ CCTCATCGAATTCGATACCAA, GTGCCAAATCAATAGAAGAA, 5′‐3′ GACTACTTGAAGGACCGGAA) and a control sequence (5′‐3′ TTCTCCGAACGTGTCACGT) from GeneChem. Following the manufacturer's instructions. The viral supernatant used to infect RAW 264.7 cells for 16 h. Then, infected cells were selected with Puromycin to establish a stable cell line. Verification of *Arrb2* knockdown was performed through western blot and RT‐PCR analysis using primers specific to *Arrb2* mRNA.

### Reagents

The following reagents from the indicated vendors were used in this study: NPD1 (Cayman Chemical, 10 010 390), ARU (Cayman Chemical, 11 817), TX14 (Anaspec, AS‐60248‐1), mouse RANKL protein (R&D systems, 462‐TEC), mouse MCSF (R&D systems, 416‐ML), anti‐IL‐10 antibody (Millipore Sigma, i5145), Forskolin (MCE, HY‐15371).

### Animals

All animal experimental procedures adhered to approved protocols the Animal Ethical and Welfare Committee of Tianjin Medical University Cancer Institute and Hospital (Approval No., NSFC‐AE‐2021146). C57BL/6 J mice (5–10 weeks) were used for all behavioral and biochemical studies. The *Gpr37* ^−/−^ mice were generously provided by Medical School of Zhejiang University. The *Arrb2^fl/fl^* mice were kindly gifted by the School of Basic Medicine, Fourth Military Medical University. Details of the mice used in the current study are listed in Table S2, Supporting Information.

### Bone Cancer Pain Model

The murine cell lines LLC1 or E0771 in the logarithmic growth phase were suspended in PBS. Mice were anesthetized with 3% isoflurane, the left lower limb at the knee joint was shaved, and the skin was sequentially disinfected with 10% iodine tincture and 75% alcohol. A superficial incision (0.5–1 cm) was made next to the knee joint to expose the patellar ligament. A 25‐gauge needle was inserted at the midpoint of the intercondylar fossa of the distal left femur into the femoral cavity. Subsequently, a 10 µL microinjection syringe was taken with 2 µL suspension of tumor cells (2 × 10 ^5^) and 2 µL of liquid gelatin sponge (for closure within the puncture site). Following needle removal, the microinjection syringe was inserted and the injection was administered slowly over a period exceeding 2 min. The surgical incision was sutured, sterilized with iodine povidone, and the mice were returned to the rearing cage for close postoperative monitoring.

### In Vivo AAV Injection into DRGs

The administration of AAV virions into DRGs was performed following established protocols.^[^ ^46^, ^47^ ^]^ In brief, 4 to 6 weeks old mice were anesthetized with isoflurane, and L3‐L4 DRGs were surgically exposed by removing the lateral processes of the corresponding vertebrae. A small incision was made in the epineurium covering each ganglion, and a glass micropipette with a fine tip was carefully inserted 100 µm into the ganglion tissue. To ensure proper sealing of the tissue around the pipette tip, a 2‐min pause was observed prior to injection. Subsequently, 1.0 µL of AAV9‐Syn‐iCre solution (GeneChem) was administered into the DRGs of *Arrb2^fl/fl^* mice at a controlled rate of 0.1 µL min^−1^ using a microprocessor‐controlled minipump. Following injection, the pipette was left in place for an additional 5 min to minimize leakage. The surgical site was then closed by suturing the overlying muscles, and the mice were placed on a 37 °C warming blanket for recovery. After a 4‐week recovery period, the mice were subjected to various assessments.

### Behavioral Tests

All the behavioral tests were conducted under strict double‐blind conditions to ensure unbiased results. Prior to testing, animals were settled in the test environment for 2 h daily over two consecutive days to reduce the impact of stress induced by unfamiliar surroundings on the experimental outcomes. Mechanical allodynia was tested using the Von‐Frey method, with filaments forces ranging from 0.02 g to 2.56 g. The hindpaw withdrawal threshold was calculated using the up‐down method, and the frequency of paw withdrawal responses is tested with 0.16 g filament. Cold allodynia was measured using nociceptive response time, where the center of the hindpaw of the affected limb was stimulated with acetone, and the time of pain‐induced paw lifting and licking was observed and recorded within 90s. Spontaneous pain was determined by flinching behaviors or guarding behaviors. While the animals were in a quiet state, the number of leg lifts and time of leg holding as guarding behavior on the tumor‐inoculated hindlimb was recorded within 2 min. Open field testing was performed to measure locomotor function. Mice were placed in the center of a 50 × 50 cm chamber, and their locomotor activity was recorded via an overhead webcam connected to a computer. The movements of the mice were automatically tracked for 20 min using VisuTrack software (Xinruan, Shanghai), and the total distance traveled and average speed during were analyzed.

### In Vivo X‐ray Radiography

X‐ray radiography was conducted via IRIS CT system (Inviscan, France) to evaluate osteolytic bone destruction in the tumor bearing femur before LLC inoculation, and on day 8, 11, and 15 post‐inoculation, respectively. The scoring criteria for bone destruction were as follows: 0, indicated normal bone for no radiographic defects; 1, indicated 1–3 radiolucent defects indicative of bone destruction; 2, indicated 3–6 radiographically visible defects along with medullary bone defects; 3, indicated medullary bone loss with partial cortical bone defects; 4, indicated complete unicortical bone defects; and 5, indicated bilateral full cortical bone defects and displaced skeletal fracture. All radiographic image quantifications were evaluated by two experimenters using a blinded method.

### In Vivo Bioluminescence Imaging

Local tumor burden was assessed by bioluminescent imaging. D‐Luciferin (Genomeditech, GM‐040611) was applied for the study. 15 min after intraperitoneal injection of D‐Luciferin (30 mg kg^−1^), bioluminescent images of tumor‐bearing leg were captured via the IVIS Lumina III system. The bioluminescence signals were analyzed using the Living Image software provided by PerkinElmer.

### Immunofluorescence Staining

Prepare frozen sections of the DRG and apply the antibody blocking solution containing 5–10% normal donkey serum or 1–2% BSA at room temperature for 2 h. For cell fluorescence, the cells were inoculated into a 24‐well plate containing climbing tablets. After cell adherence, they were fixed with 4% paraformaldehyde for 10–15 min and rinsed with PBS three times. Triton X‐100 permeates cells and enhances cell membrane permeability. After discarding the blocking solution, the sections were incubated overnight at 4 °C in a humidified chamber with primary antibodies including GPR37 (Abcam, ab218134, 1:500), NeuN (Proteintech, 26 975, 1:500), CGRP (Abcam, ab36001,1:3000), NF200 (Proteintech, 18 934, 1:500), IB4 (vectorlabs, FL‐1201, 20 µg mL^−1^), β‐arrestin 2 (CST, 3857, 1:500). Subsequently, the primary antibody was discarded, and the sections were washed and incubated in secondary antibody (with optional DAPI) including FITC‐conjugated affinipure donkey anti‐goat IgG (Protentech, SA00003, 1:100) and Cy3‐ conjugated affinipure goat anti‐rabbit IgG (Protentech, SA00009, 1:100) at 37 °C in the dark for 2 h. Following washing steps and mounting, images were acquired under a confocal laser scanning microscope, and further analyzed using Image J (NIH).

### Histological Staining of Tumor‐Bearing Femora

Following deep anesthesia of the mice, the femur was extracted post intracardiac perfusion with PBS and 4% paraformaldehyde (PFA), and subsequently fixed with 4% PFA at 4 °C for 48 h. Decalcification of the femur was achieved using 10% EDTA for 10 days, followed by paraffin embedding post ascending gradient dehydration with 30–100% ethanol. Sections of trabecular bone, 5 µm in thickness, were obtained from the distal femur and stained with TRAP or GPR37 (Abcam, ab218134, 1:500). The TRAP kit (Biossci, BP088) was used. Bone static histomorphometric analysis was conducted using Image J based on images captured with a Leica Q500MC microscope. The count of osteoclasts (within 1500 µm of the proximal end of the growth plate) was determined by dividing the number of osteoclasts by the perimeter of the trabecular bone where they were situated.

### In Situ Hybridization (RNAscope)

DRG frozen sections (12 µm) were prepared, mounted on glass slides, and air‐dried overnight at room temperature (24–26 °C). The slides were washed with 1× PBS for 5 min and fixed in 4% paraformaldehyde for 15 min. After gradient dehydration in 50%, 70%, and 100% ethanol, specimens were pre‐treated with H2O2 (ACD, 2 024 994) for 10 min at room temperature. The sections were incubated with Protease III (ACD, 2 024 996) at 40 °C in a water bath (HybEZ Oven) for 30 min, followed by hybridization with the GPR37 probe (Bio‐Techne, 24101A) for 2 h. Signal amplification was performed sequentially with AMP1 (ACD, 2 025 226), AMP2 (ACD, 2 025 227), and AMP3 (ACD, 2 025 228), followed by channel detection using HRP‐C1 (ACD, 2 025 229), fluorophore‐conjugated tyramide (ACD, PG‐323271, 520 nm, 1:1000), and blocker (ACD, 2 025 232). All staining steps were carried out at 40 °C in a water bath. For Nissl counterstaining, sections were washed with 1× PBS, incubated with Nissl stain (Invitrogen, 2 291 540, 435/455 nm, 1:500) in the dark for 30 min, and immersed in 1× PBS at 4 °C for 12 h. Coverslips were mounted with an anti‐fade medium. Images were captured using an Olympus FV3000 confocal microscope. Quantitative analysis with ImageJ included the proportion of GPR37 mRNA positive neurons among Nissl+ neurons and the density of GPR37 mRNA‐positive puncta per unit area in the DRG. For each mouse, 4–5 sections were used for staining and analysis.

### Elisa

Elisa kits including NPD1 (Biomatik, EKF58060), CTX‐I (CUSABIO, CSB‐E11224 h), IL‐10 (CUSABIO, CSB‐E04594 m) were obtained and applied. The samples of human or murine serum or cell culture supernatant were added and tested as per the manufacturer's instructions. For the obtainment and detection of CSF, mice were anesthetized via inhalation administration of isoflurane and placed on a stereotactic frame. Cerebrospinal fluid was collected from the cisterna magna under a dissection microscope using a glass capillary.^[^ ^48^ ^]^ Then level of NPD1 in CSF was measured according to the manufacturer's instructions.

### Flow Cytometry

Tumor tissues were excised from mice and mechanically minced into small fragments. These fragments were enzymatically digested in a solution containing 1 mg mL^−1^ collagenase D and 0.1 mg mL^−1^ DNase I, prepared in RPMI‐1640 medium, at 37 °C for 30 min with gentle agitation. The resulting cell suspensions were filtered through a 70 µm cell strainer to obtain single‐cell suspensions. Cells were then washed and resuspended twice with PBS containing 2% FBS. For immunostaining, cells (1 × 10⁶ per sample) were incubated with the following fluorescently conjugated antibodies for 30 min at 4 °C in the dark: CD45‐BB700 (BD, 566 439), CD11b‐R718 (BD, 567 469), F4/80‐BUV395 (BD, 565 614), CD86‐BV421 (BD, 564 198), and CD206‐PE (BD, 568 273). After staining, cells were washed twice with FACS buffer and resuspended in 300 µL of the same buffer. Flow cytometric data were acquired using a Beckman Coulter CytoFLEX LX. The gating strategy included the following steps: single cells were identified by forward scatter (FSC‐A versus FSC‐H), leukocytes were gated as CD45⁺ cells, and macrophages were identified as CD45⁺CD11b⁺F4/80⁺. Further characterization of macrophage subtypes was performed by identifying M1 like macrophages as CD86⁺CD206⁻ and M2 like macrophages as CD206⁺CD86⁻.

### Real‐Time PCR

Total RNA was extracted from tissue or cell samples following the manufacturer's instructions using a commercial RNA extraction kit (TIANGEN, DP451). First‐strand cDNA was synthesized from 1 µg of total RNA using a reverse transcription kit (GenStar, A230‐10). Quantitative PCR was performed using a SYBR Green Master Mix (US EVERVRIGHT, S2024L) on a real‐time PCR system. The reaction was conducted in a 20 µL volume. Relative expression levels of target genes were calculated using the 2⁻ΔΔCT method, normalizing to the expression of a housekeeping gene (GAPDH). For each sample, reactions were performed in triplicate, and the average CT value was used for analysis. For Gpr37: forward sequence 5′‐3′, CCATGAGTTGACTAAGAAGTGGC; reverse sequence 5′–3′, GGAAGCGATCTATGCACAGTGC).

### Western Blot

Tissues or cells were lysed in RIPA buffer (Solarbio, R0010) supplemented with protease and phosphatase inhibitors (Selleck, B14001 and B15001, EDTA‐free). The lysates were centrifuged at 12 000 × g for 15 min at 4 °C to remove debris. Supernatants were collected as total protein extracts, and protein concentration was determined using a BCA protein assay kit (Thermo Fisher Scientific, 23 227). The membranes were incubated overnight at 4 °C with the primary antibodies GAPDH (Ray Antibody, 1:5000), GPR37 (abcam, ab218134, 1:1000), β‐arrestin 2 (CST, 3857, 1:1000), diluted in TBST containing 5% BSA. Band intensity was quantified using ImageJ software. Protein expression levels were normalized to the loading control (GAPDH).

### Bone Marrow‐Derived Macrophages from Mice Culture

Mice were euthanized and the femur was carefully removed and placed on ice. The muscles surrounding the femur were removed as gently as possible, the distal epiphysis was isolated from the femoral stem, the proximal femur was excised, and a 25‐gauge needle was inserted into the femoral cavity to inject 1 mL of cold PBS or α‐MEM (Gibco, Thermo Fisher Scientific) for the bone marrow to be emptied into a 1.5 mL centrifuge tube. Bone marrow cells were harvested by centrifugation at 800 g for 5 min at 4 °C, and the isolated bone marrow cells were cultured overnight in α‐MEM medium supplemented with 10% FBS and 1% antibiotics. The suspended cells were then collected and cultured with 20 ng mL^−1^ MCSF for 3 days, and the adherent cells were BMDM.

### In Vitro Induction of Osteoclastogenesis and TRAP Staining

The RAW 264.7 cells were incubated with 35 ng mL^−1^ RANKL for six days. For BMDMs, they were induced by 35 ng mL^−1^ RANKL and 20 ng mL^−1^ MCSF. Culture medium and applied reagents were changed every 3 days. After 6 or 7 days of incubation, the cells were fixed by 4% PFA and stained with TRAP staining solution (TRAP kit, Servicebio, G1050). The osteoclasts were identified as TRAP‐positive multinucleated cells (3 or more nuclei) under light microscopy. Image J software was employed to analyze the number and area ratio of osteoclasts in random fields of view.

### Whole‐Cell Patch Clamp Recording in Cultured DRG Neurons

L3‐L5 DRG segments were extracted from 7–10 weeks old mice on day 14 after LLC inoculation and digested using a solution of collagenase (1.25 mg mL^−1^) or dispase‐II (2.4 U mL^−1^) HBSS at 37 °C for 90 min, followed by a 10‐min incubation in 0.25% trypsin. The cells were then mechanically dispersed, plated onto glass coverslips coated with 0.5 mg mL^−1^ poly‐D‐lysine, and cultured in Neurobasal medium supplemented with 10% FBS, 2% B27, and 1% antibiotics at 37 °C in 5% CO_2_ for 24 h. Whole‐cell patch clamp recordings were performed on small‐sized DRG neurons (< 25 µm in diameter) at room temperature using an Axopatch‐700B amplifier with a Digidata 1440A. To evaluate the excitability of DRG neurons, the current clamp mode was established to record action potentials (APs). Pipette resistance was 4–6 MΩ for the whole‐cell recording, and it was filled with a recording solution containing (in mM): 126 K‐gluconate, 10 NaCl, 1 MgCl_2_, 10 EGTA, 10 HEPES and 2 Na‐ATP (adjusted to pH 7.4 with KOH). The external solution contained (in mM): 140 NaCl, 5 KCl, 2 CaCl_2_, 1 MgCl_2_, 10 HEPES, 10 glucose, adjusted to pH 7.4 with NaOH. The APs were triggered by current injection steps from 0–130 pA, with increments of 10 pA over 600 ms. For the rheobase recording, current injection for action potential induction starts from 0 pA and increases 10 pA per step for 30 ms.

### Whole‐Cell Patch‐Clamp Recording in Spinal Cord Slices

The mice (male, 5–6 weeks old) with BCP were anaesthetized. The lumbosacral spinal cord was gently removed and put in an ice‐cold dissection solution (in mM): 240 Sucrose, 25 NaHCO_3_, 2.5 KCl, 1.25 NaH_2_PO_4_, 0.5 CaCl_2_ and 3.5 MgCl_2_, equilibrated with 95% O_2_ and 5% CO_2_. After spinal extraction under anesthesia, animals were sacrificed by decapitation. Transverse spinal slices (400 µm) were cut using a vibrating microslicer. The slices were incubated at 34 °C for at least 60 min in artificial cerebrospinal fluid (ACSF, in mM): 126 NaCl, 3 KCl, 1.3 MgCl_2_, 2.5 CaCl_2_, 26 NaHCO_3_, 1.25 NaH_2_PO_4_ and 11 glucose, equilibrated with 95% O_2_ and 5% CO_2_. The slices were then placed in a recording chamber and perfused at a flow rate of 2–4 mL min^−1^ with ACSF, which was saturated with O_2_ at room temperature. Whole‐cell voltage‐clamp recordings were made. The patch‐pipette solution that was used to record sEPSCs contained (in mM): 135 K‐gluconate, 5 KCl, 0.5 CaCl_2_, 2 MgCl_2_, 5 EGTA, 5 HEPES, 5 Mg‐ATP (pH 7.3 adjusted with KOH). The patch pipettes had a resistance of 6–8 M. sEPSCs recordings were made at a holding potential of −70 mV. Signals were acquired using an Axopatch 700B amplifier. The data were stored and analyzed with a personal computer using pCLAMP 10.3. sEPSC events were detected and analyzed using Mini Analysis Program ver. 6.0.3.

### Single‐Cell RNA‐Seq Database Analysis

Publicly available single‐cell RNA sequencing (scRNA‐seq) dataset (GSE202051) were analyzed to investigate the role of GPR37 in macrophages. Dimensionality reduction and unsupervised clustering were performed using the t‐distributed stochastic neighbor embedding (t‐SNE) algorithm to identify and visualize distinct cell populations, with cell type annotations based on canonical marker genes. Macrophage populations were further extracted and re‐clustered into subclusters to explore heterogeneity within this cell type. Macrophages were subsequently categorized into GPR37‐positive and GPR37‐negative groups based on GPR37 expression levels, followed by differential expression analysis. Gene Ontology (GO) analysis was performed to identify biological processes, molecular functions, and cellular components associated with these differentially expressed genes. Enrichment analysis of these genes was conducted to determine significantly associated pathways. Additionally, pathway enrichment scores and the expression patterns of key genes, such as β‐arrestin, IL10/IL10RA, were calculated and mapped onto macrophage subclusters to explore their relationships with GPR37 expression. All bioinformatics analyses were performed using Seurat and SCP packages.

### Clinical Study

The retrospective clinical study was approved by the Ethics Committee of Tianjin Medical University Cancer Institute and Hospital (Approval No. bc2021036) and informed consent was obtained from participants or next of kin. Seventy‐nine patients with bone metastasis from lung cancer or breast cancer in Tianjin Medical University Cancer Hospital from February 2015 to February 2021 were enrolled. These patients were pathologically diagnosed as non‐small cell lung cancer or breast cancer, with complete clinical records including pain intensity scores, imaging data, and plasma specimens. The exclusion criteria included: history of previous tumor treatment, history of opioid application, history of bone metabolic diseases, and history of neurological and psychiatric diseases.

### Statistical Analysis

The sample size calculation for this study was based on our previous studies on such experiments.^[^ ^5^, ^49^, ^50^ ^]^ Statistical analysis and graphical representations were carried out using GraphPad Prism 6.0. All data were presented as mean ± standard error (SEM) or median with interquartile range. Statistical comparisons of molecular and behavioral parameters were conducted utilizing two‐tailed Student's *t‐*test, One‐Way ANOVA or Two‐Way repeated ANOVA with post‐hoc Bonferroni test. Rates were compared using Fisher's exact test. Clinical data were analyzed with Mann‐Whitney test and Person correlation test. *p* < 0.05 was considered statistically significant.

## Conflict of Interest

The authors declare no conflict of interest.

## Author Contributions

K.W., Y.Z., and R.S. contributed equally to this work. K.W., Y.L., and Y.Y. conceived and designed the experiments. K.W., Y.Z., R.S., L.Y., H.T., S.W., Y.‐F.Z. and C.J. conducted the experiments and analyzed the data. K.W., Y.Z., and R.S. performed the clinical investigation. K.W. and Y.L. wrote the manuscript, while C.J. and Y.Y. revised the manuscript. All authors checked and approved the final manuscript.

## Supporting information



Supporting Information

## Data Availability

Data that support the findings of this study are available from the corresponding author upon reasonable request.
