# Supplementary material for: GPR37 Activation Alleviates Bone Cancer Pain via the Inhibition of Osteoclastogenesis and Neuronal Hyperexcitability
Source: Adv Sci (Weinh). 2025 Feb 18;12(14):2417367. doi: 10.1002/advs.202417367 (PMC11984854; doi:10.1002/advs.202417367)
Supplement: Supplementary file 1 — Supporting Information [file ADVS-12-2417367-s001.docx]

**Supporting Information**

**Title:** GPR37 Activation Alleviates Bone Cancer Pain via the Inhibition of Osteoclastogenesis and Neuronal Hyperexcitability

**Authors:** *Kaiyuan Wang,* Yongfang Zhang, Ruichen Shu, Limei Yuan, Huifang Tu, Shengran Wang, Bo Ni, Yi-Fan Zhang, Changyu Jiang, Yuhui Luo,* and Yiqing Yin**

**Correspondence to:** Kaiyuan Wang ([kywang@tmu.edu.cn](mailto:kywang@tmu.edu.cn))

Yuhui Luo ([luosteven2000@aliyun.com](mailto:luosteven2000@aliyun.com))

Yiqing Yin ([yinyiqing@tmu.edu.cn](mailto:yinyiqing@tmu.edu.cn))

**
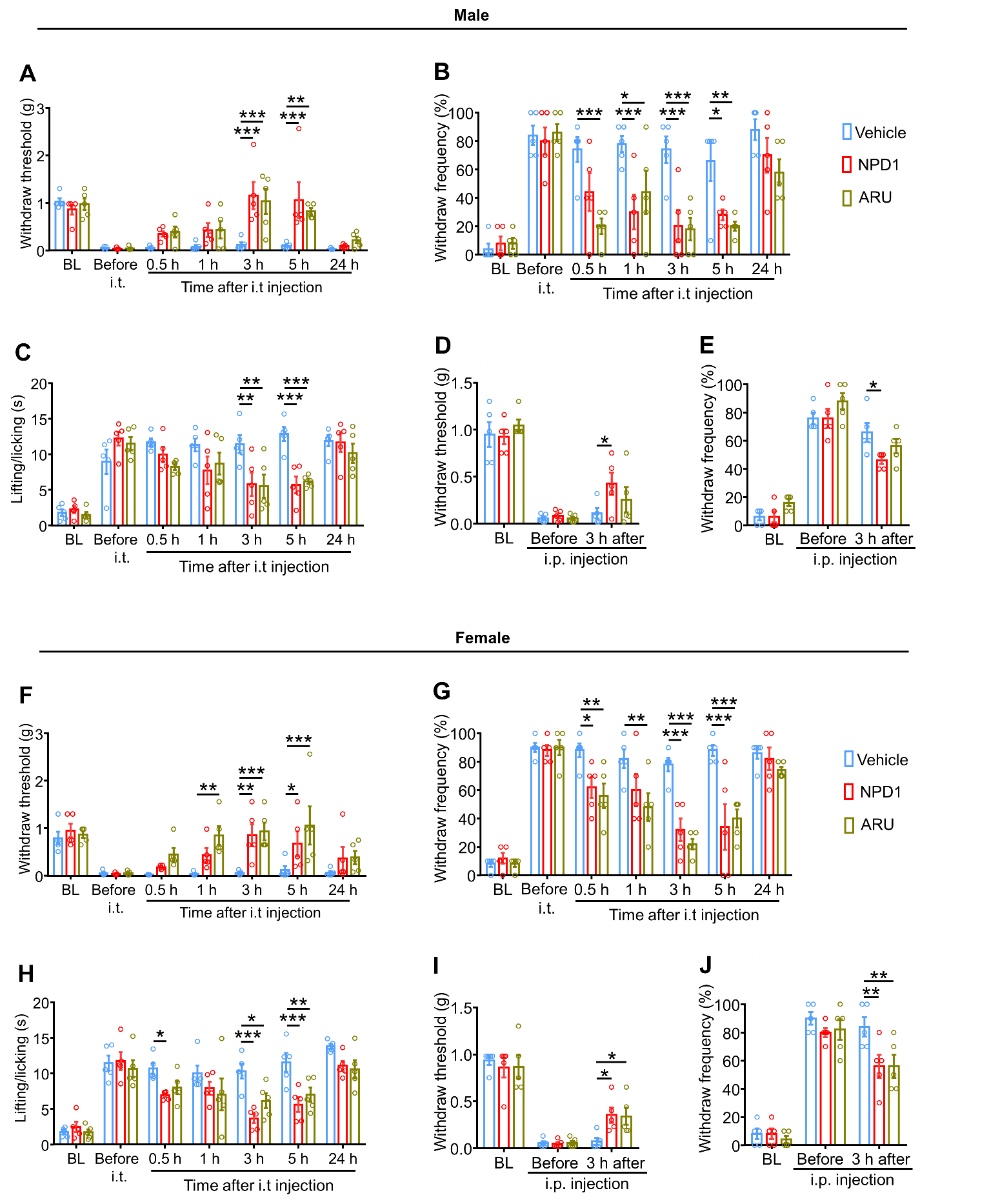
**

**Figure S1. GPR37 agonists reduce bone cancer pain in both male and female mice. A-B** Von Fery testing to detect cancer induced mechanical allodynia, as assessed by withdrawal threshold (**A**) and withdrawal frequency (**B**) in male mice i.t. injected with vehicle, NDP1 (200 ng) or ARU (20 μg) on day 11 after LLC inoculation. n = 5 male mice/group. **C** Acetone response test to determine cold allodynia after the indicated treatment. n = 5 male mice/group. **D-E** Change of mechanical allodynia through von Frey testing after i.p. injection with vehicle, NPD1 (25 μg/kg) or ARU (25 mg/kg) on day 11 after tumor inoculation. n = 5 male mice/group. **F-G** Cancer induced mechanical allodynia, as measured by withdrawal threshold (**F**) and withdrawal frequency (**G**) in female mice with indicated treatment via Von Fery testing. n = 5 female mice/group. **H** Acetone response test to detect cold allodynia after i.t. injection of vehicle, NPD1 or ARU. n = 5 female mice/group. **I-J** Change of mechanical allodynia through von Frey testing after the indicated i.p. treatment on day 11 after tumor inoculation. n = 5 female mice/group. All data are displayed as mean ± SEM, and analyzed using repeated-measures two-way ANOVA with Bonferroni’s post-hoc test, **P*<0.05, ** *P*<0.01, ****P*<0.001.

**
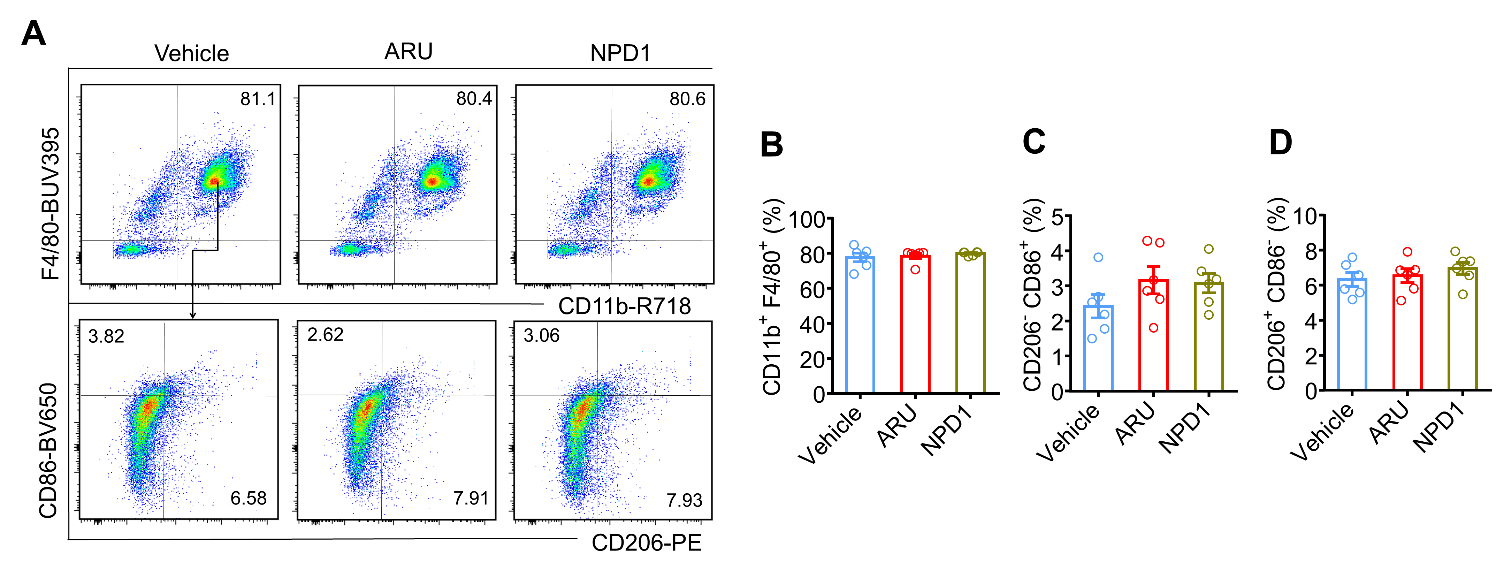
**

**Figure S2. Comparison of tumor associated macrophage after ARU or NPD1 treatment.** **A** Representative flowcytometry images of tumor associated macrophage (TAM) in tumor tissue from BCP mice on day 11 after tumor inoculation. **B**-**D** Quantification and comparison of the percentage of total TAMs (CD11b^+^ F4/80^+^), M1 like TAM (CD206^-^ CD86^+^) or M2 like TAM (CD206^+^ CD86^-^) among the vehicle, NPD1 or ARU treatment. n = 6 mice per group. All data are expressed as the mean ± SEM, and analyzed using one-way ANOVA with Bonferroni’s post-hoc test.

**
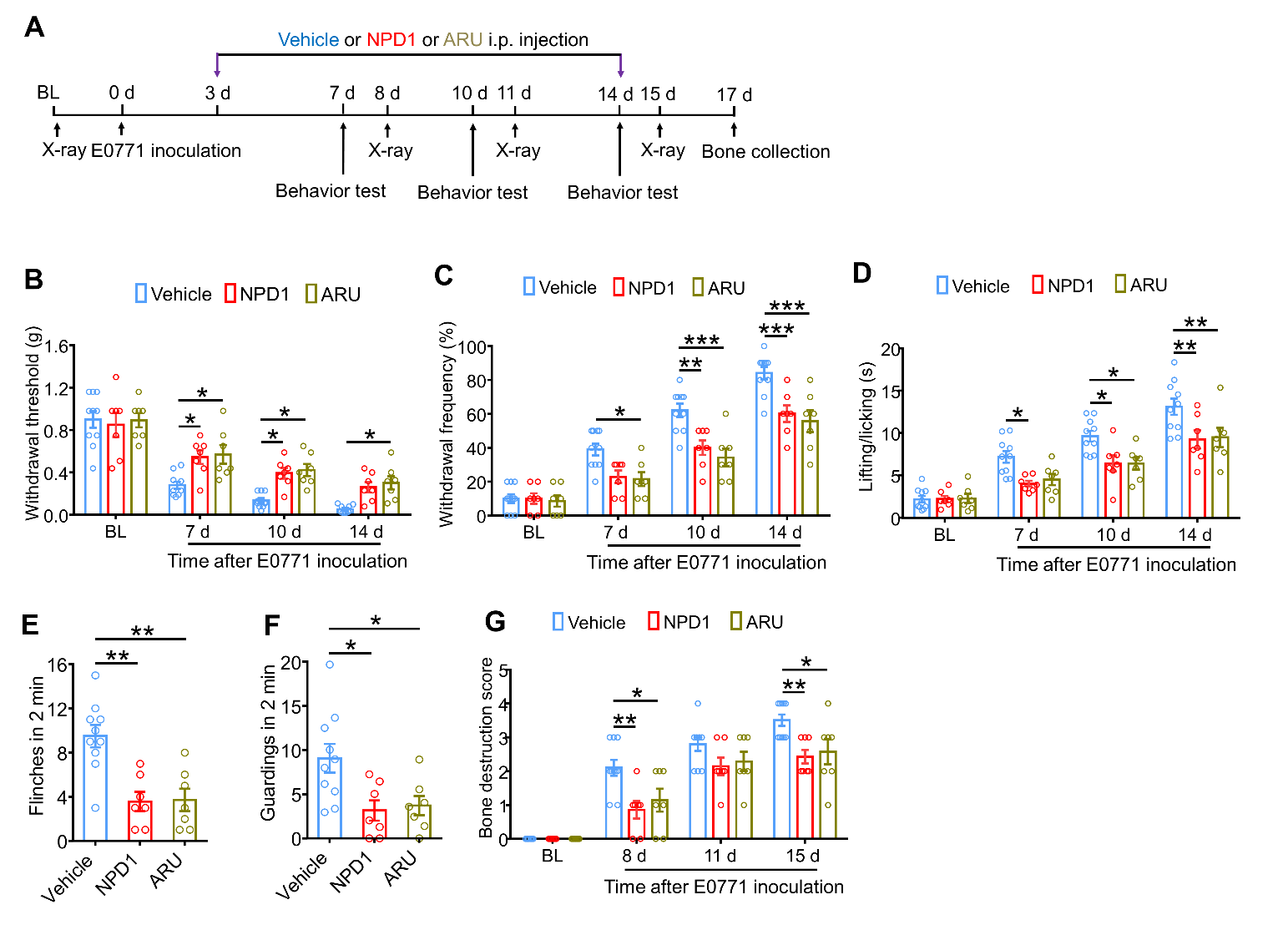
**

**Figure S3. GPR37 agonist confer protection in breast cancer induced bone pain and bone destruction. A** Diagram for experimental design. **B-C** Von Frey testing to measure mechanical allodynia through withdrawal threshold (**B**) and withdrawal frequency (**C**) in mice treated with vehicle, NPD1 or ARU on day 7, 10 and 14 after E0771 inoculation. **D** Cold allodynia via acetone response test in mice with indicated treatment. **E-F** Observation of spontaneous pain as shown by flinching (**E**) or guarding behaviors (**F**) in vehicle, NPD1 or ARU treated mice on day14 after E0771 implantation. **G** Change of bone destruction score in tumor bearing femora from X-ray testing on day 8, 11 and 15 after E0771 inoculation. n = 7-10 mice/group for the above panels. All data displayed represent the mean ± SEM, and are analyzed using repeated-measures two-way ANOVA with Bonferroni’s post-hoc test (B, C, D, G), or one-way ANOVA with Bonferroni’s post-hoc test (E, F), **P*<0.05, ** *P*<0.01, ****P*<0.001.


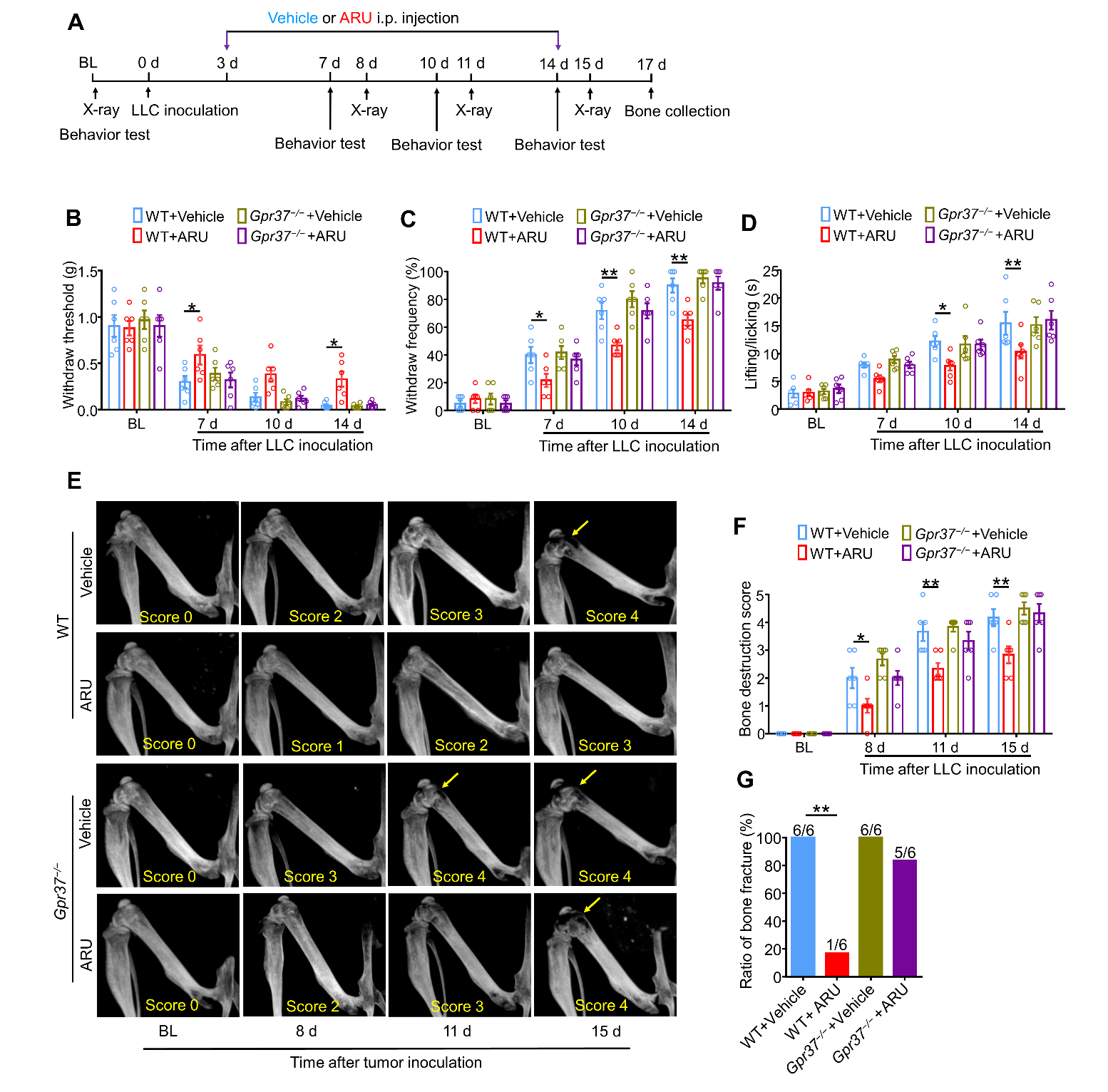


**Figure S4. GPR37 mediates the pain relief and bone protection from ARU treatment. A** Study design to detect the protective effect of i.p. injection of ARU (25 mg/kg) in WT or *Gpr37^−/−^* mice. **B-C** Mechanical allodynia measured by withdrawal threshold (**B**) and withdrawal frequency (**C**) on day 7, 10 and 14 after tumor inoculation. **D** Cold allodynia via acetone response testing. **E-F** Representative X-ray images (**E**) and quantification of bone destruction score (**F**) in WT or *Gpr37^−/−^* mice applied with vehicle or ARU (25 mg/kg) on day 8, 11 and 15 post LLC inoculation. Bone destruction score is indicated in each image and arrows show bone lesions with scores over 3. **G** Comparison of ratio of bone fracture in tumor bearing femora on day 17 after LLC implantation. n = 6 mice/group. All data indicate the mean ± SEM, and are analyzed using repeated-measures two-way ANOVA with Bonferroni’s post-hoc test (B, C, D, F), or two-sided Fisher’s exact test (G), **P*<0.05, ***P*<0.01.


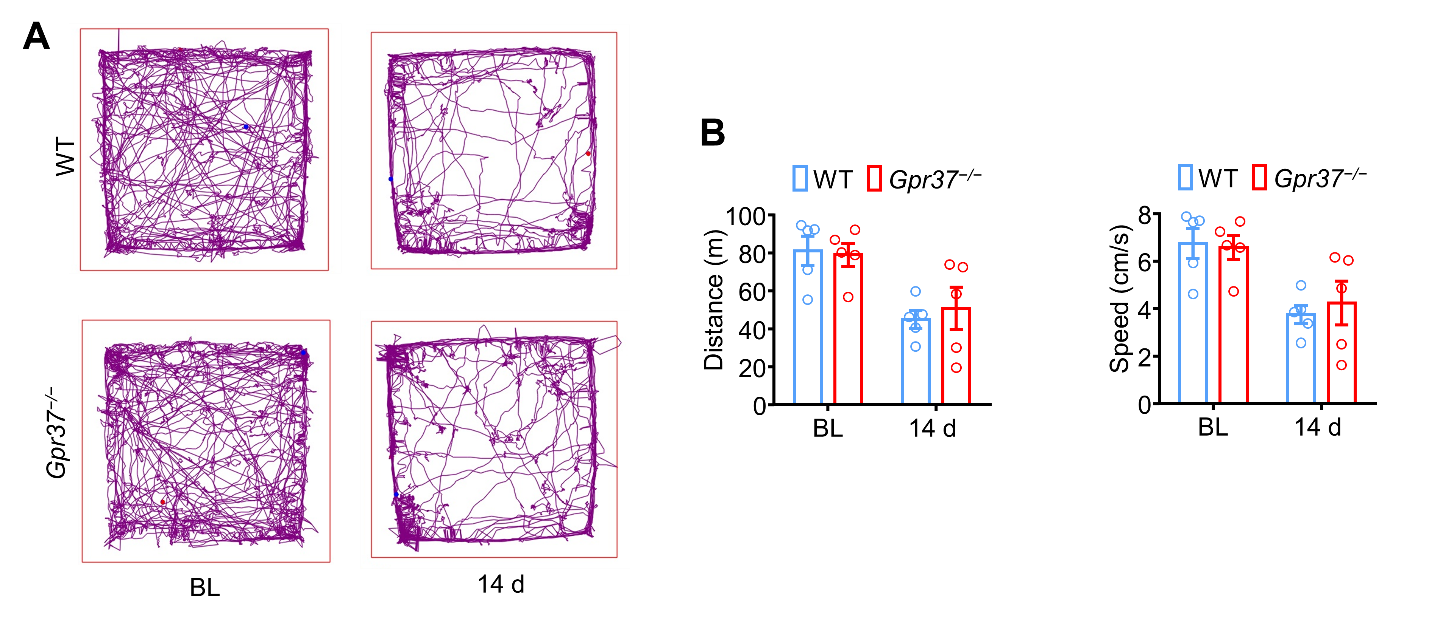


**Figure S5. Effect of GPR37 on locomotor function.** **A-B** Open field testing detecting distance traveled and mean speed over a 20 min duration in WT mice or *Gpr37*^−/−^ mice at baseline or 14 days after LLC inoculation. **A** Representative trace. **B** quantification for (**A**). n = 5 mice/group for the above panels. All data are expressed as mean ± SEM, and analyzed using repeated-measures two-way ANOVA with Bonferroni’s post-hoc test.

**
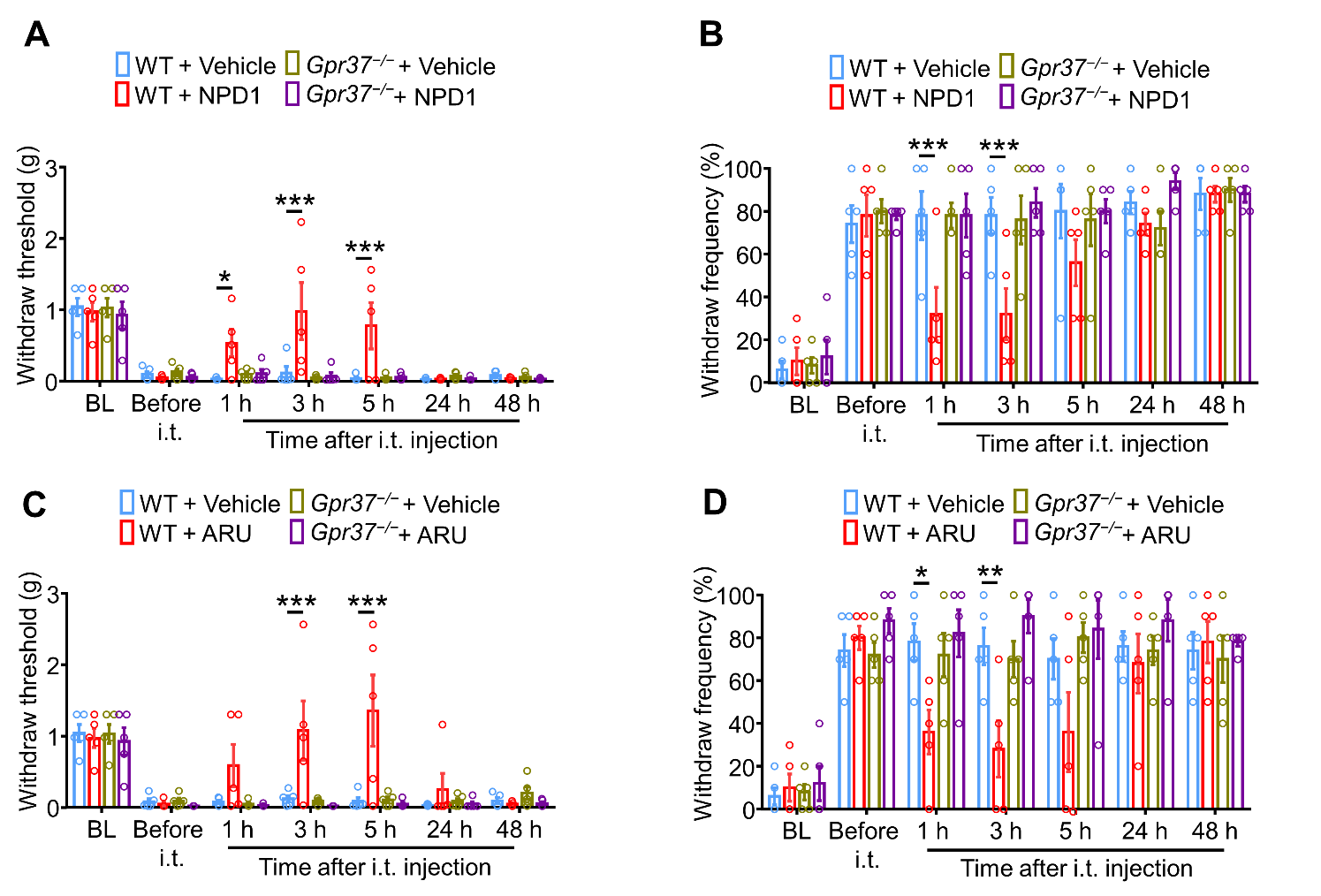
**

**Figure S6. Instant analgesic effect of NPD1 and ARU is GPR37 dependent.** **A-B** Mechanical allodynia measured by withdrawal threshold (**A**) and withdrawal frequency (**B**) in WT or *Gpr37^−/−^* mice after i.t. injection of vehicle or NPD1 on day 11 after LLC inoculation. n = 5 mice per group. **C-D** Mechanical allodynia detected by withdrawal threshold (**C**) and withdrawal frequency (**D**) in WT or *Gpr37^−/−^* mice after i.t. injection of vehicle or ARU on day 11 after LLC inoculation. n = 5 mice per group. Data displayed represent the mean ± SEM, and are analyzed using repeated-measures two-way ANOVA with Bonferroni’s post-hoc test, **P*<0.05, ** *P*<0.01, ****P*<0.001.


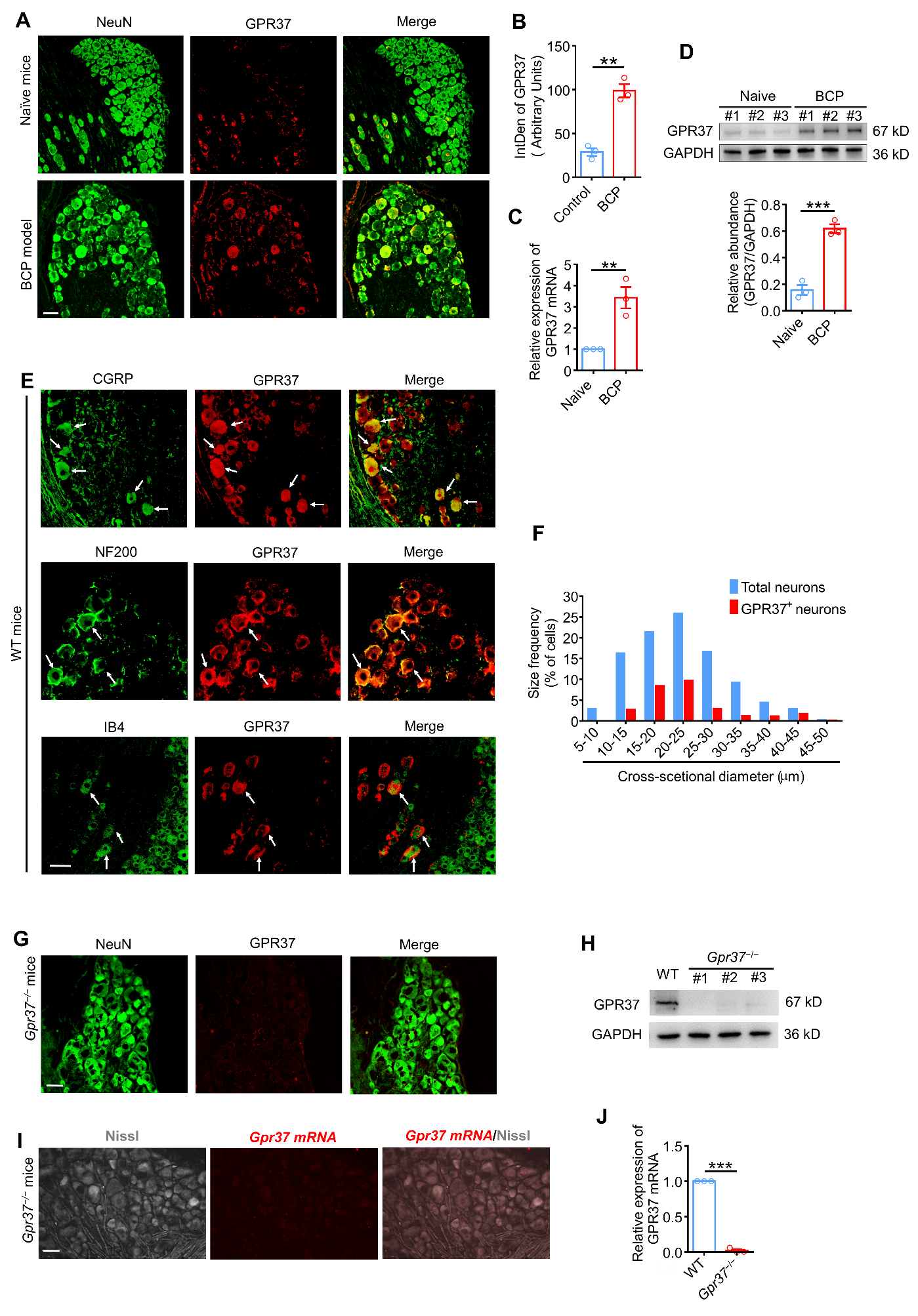


**Figure S7. Increased expression of GPR37 in DRG neurons from tumor bearing mice.** **A** Representative immunostaining images showing GPR37 expression in L3-L5 DRG neurons from naïve mice or mice with bone cancer pain. Scale bar, 50 μm. **B** Quantification for panel (**A**). n = 3 mice per group. **C** RT-PCR showing increased expression of GPR37 mRNA in DRG neurons from BCP mice. n = 3 mice per group. **D** Western blot showing the expression of GPR37 in DRG neurons from naïve mice or BCP mice**.** Top, western blot bands. Lower, Quantification. n = 3 mice per group. **E** Double immunostaining of GPR37 with CGRP, NF200 and IB4 in DRG neurons from mice with BCP. Arrows indicate the co-expression. Scale bar, 50 μm. **F** The size distribution of GPR37^+^ neurons and total neurons in mouse DRGs. A total of 1,032 neurons from 3 WT mice with BCP were analyzed. **G** Representative immunostaining images showing the absent GPR37 expression in L3-L5 DRG neurons from *Gpr37*^−/−^ mice with BCP. Scale bar, 50 μm. **H** Western blot detecting the expression of GPR37 in WT or *Gpr37*^−/−^ mice with BCP. **I** RNAscope showing the absent expression of *Gpr37* mRNA in L3-L5 DRG neurons from *Gpr37*^−/−^ mice with BCP. Scale bar, 40 μm. **J** RT-PCR showing decreased expression of GPR37 mRNA in DRG neurons from WT or *Gpr37*^−/−^ mice with BCP. n = 3 mice per group. Data displayed represent the mean ± SEM, and are analyzed with two-tailed Student’s t-test, ** *P*<0.01, *** *P*<0.001.


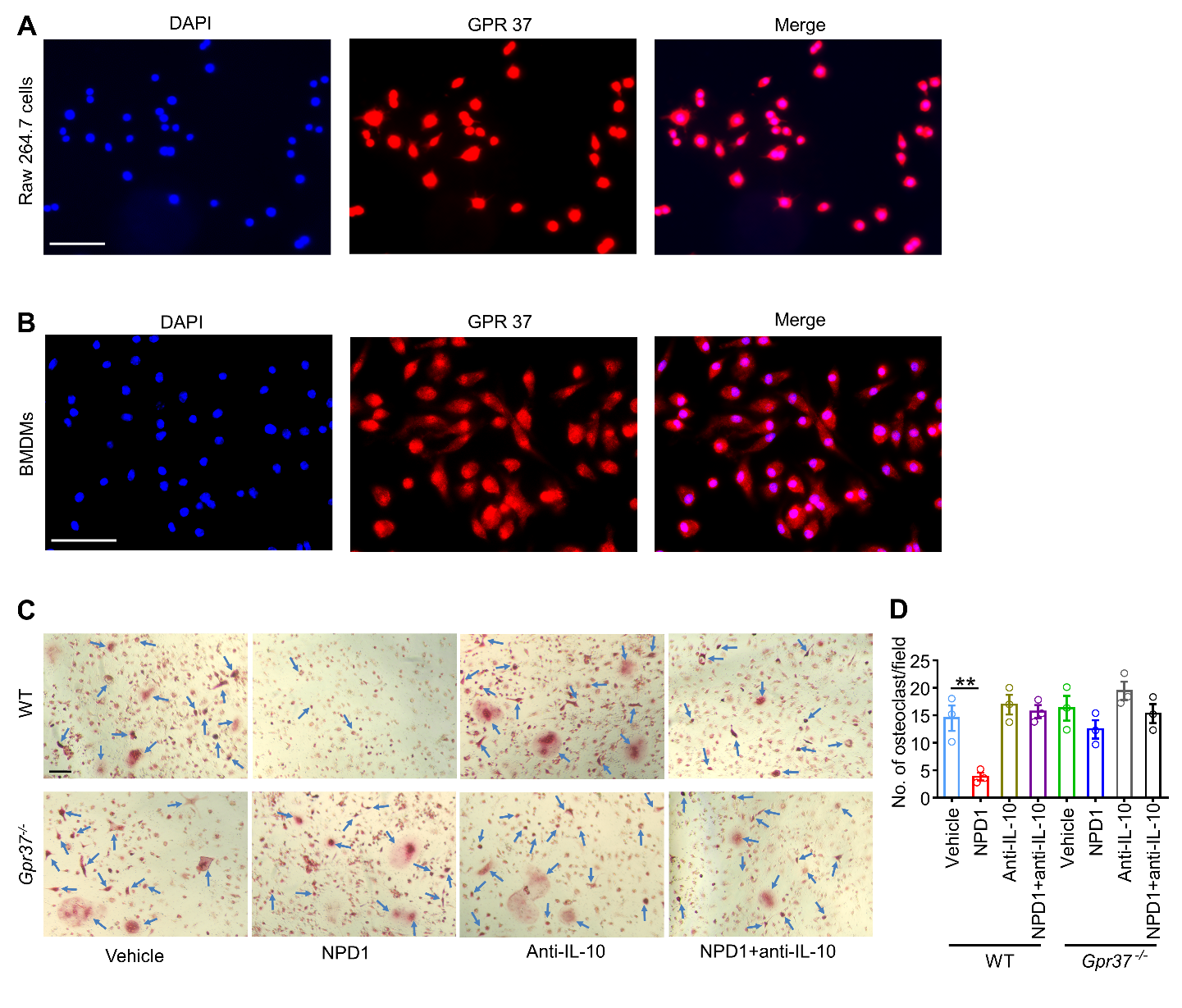


**Figure S8. High expression of GPR37 in Raw 264.7 cells and BMDMs**. **A** Double immunostaining of DAPI and GPR37 in Raw 264.7 cells. Scale bar, 50 µm. **B** Double immunostaining of DAPI and GPR37 in bone marrow derived macrophages (BMDMs). Scale bar, 50 µm. **C-D** TRAP staining for osteoclasts differentiated from BMDM cells from WT mice or *Gpr37^−/−^* mice, each treated with vehicle, NPD1 (100 nM), anti-IL-10 antibody (1 μg/mL) or NPD1 + anti-IL-10 antibody. RANKL: 35 ng/mL, MCSF: 20 ng/mL. **C** Representative images of TRAP staining. Arrows indicate TRAP^+^ osteoclasts. Scale bar, 100 µm. **D** Quantification for (**C**), n = 3 independent cultures. Data are presented as the mean ± SEM, and analyzed using one-way ANOVA with Bonferroni’s post-hoc test, ** *P*<0.01, *** *P*<0.001.


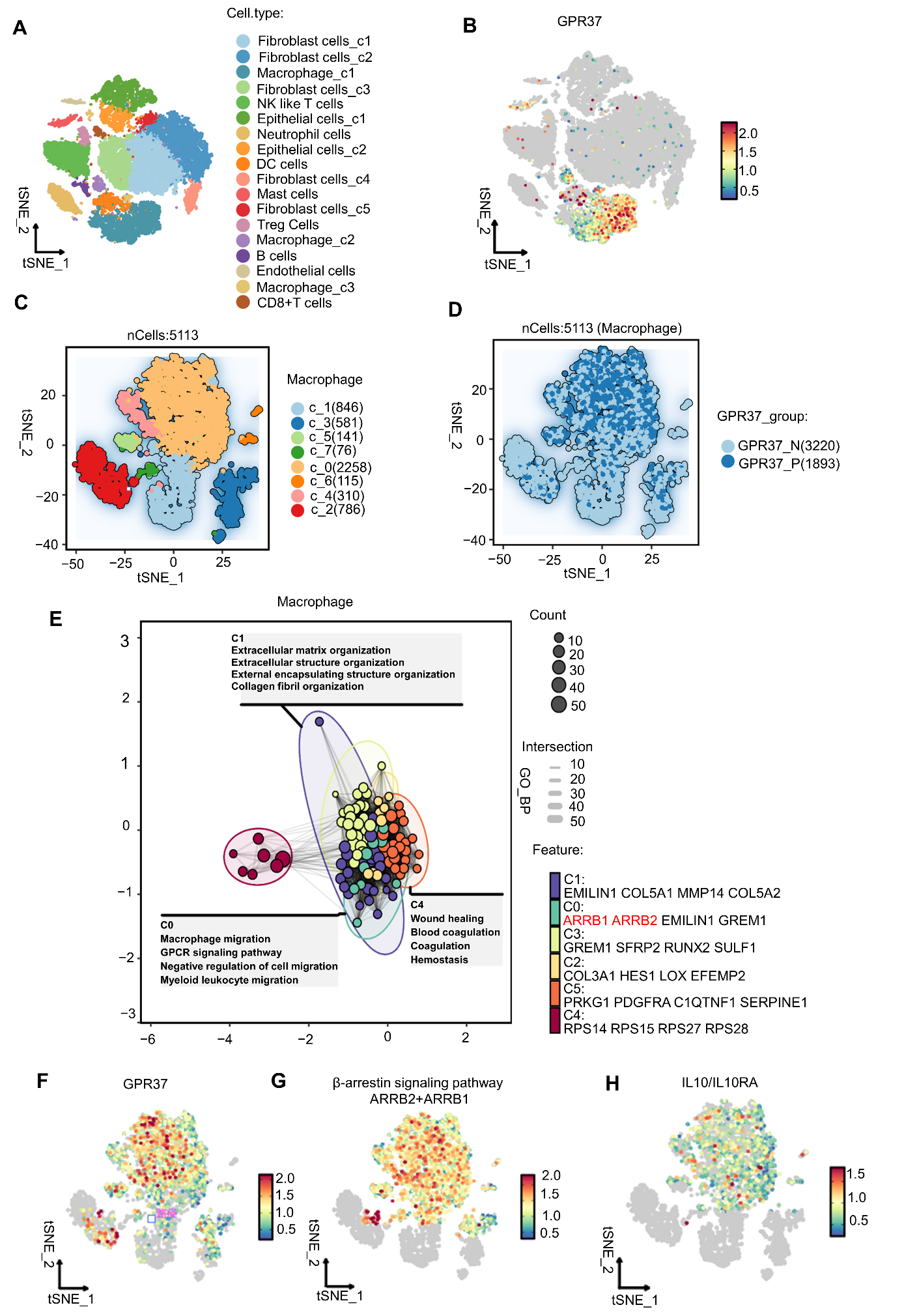


**Figure S9. Single-cell RNA sequencing data analysis verifying the role of β-arrestin in GPR37-mediated IL-10 regulation. A** t-SNE plot showing the distribution of various cell subpopulations within the single-cell dataset (GSE202051). **B** t-SNE plot illustrating the expression levels of GPR37 across different cell populations. **C** t-SNE plot displaying the distribution of macrophage subclusters following dimensionality reduction and clustering. **D** t-SNE plot showing the classification of macrophages into GPR37-positive and GPR37-negative groups based on GPR37 expression. **E** GO pathway enrichment analysis of differentially expressed genes between GPR37-positive and GPR37-negative macrophages. **F** t-SNE plot presenting the expression levels of GPR37 in macrophages. **G** t-SNE plot showing the distribution and expression of ARRB 2 and ARRB1 (genes of β-arrestin pathway) in macrophages. **H** t-SNE plot displaying the expression levels of the IL-10RA gene in macrophages.

**
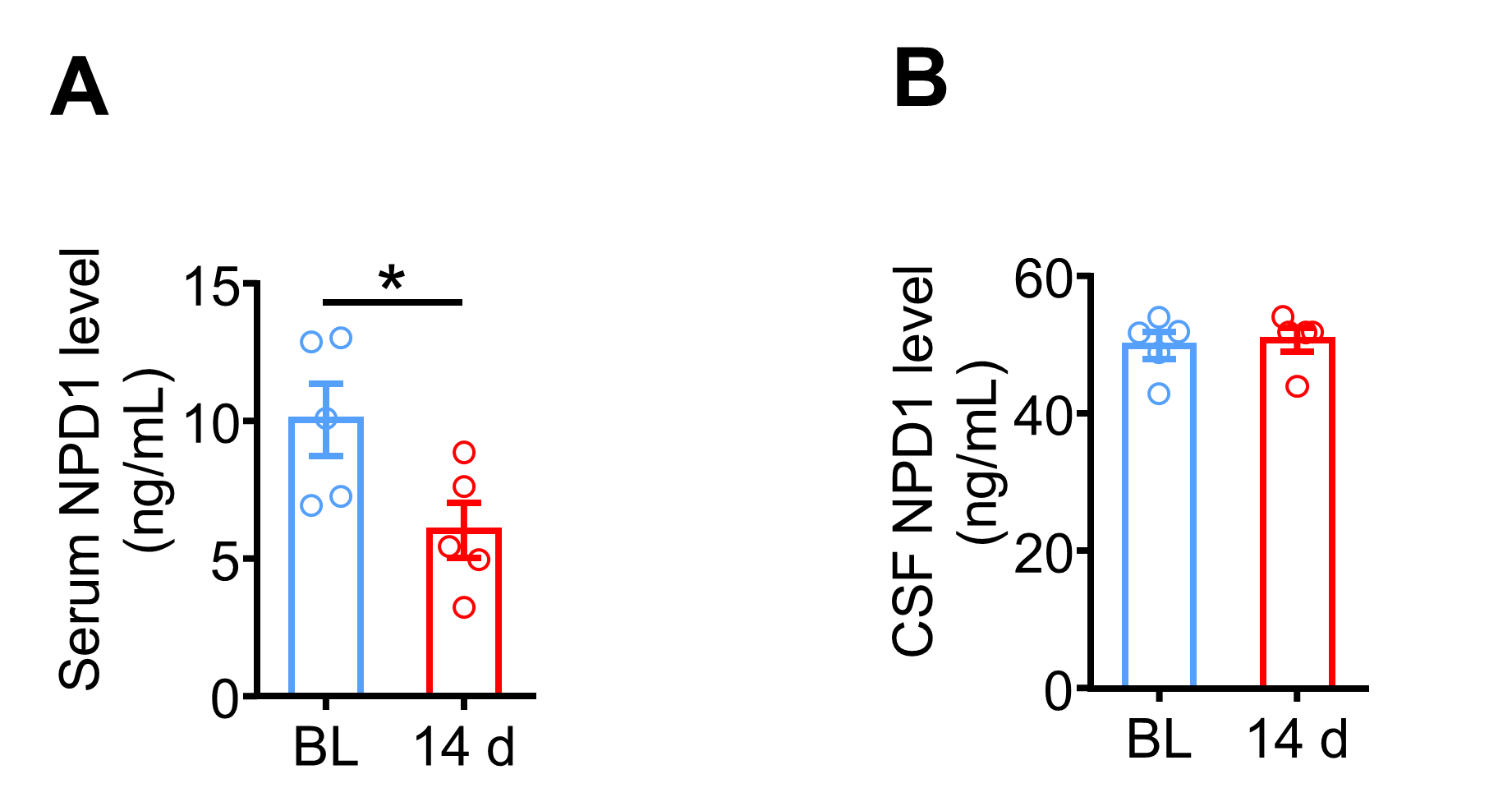
**

**Figure S10. Measurement of NPD1 level in serum or cerebrospinal fluid. A** Change of serum NPD1 level from mice at baseline or 14 days after LLC inoculation. n = 5 mice per group. **B** Change of endogenous NPD1 level in cerebrospinal fluid from mice at baseline or 14 days after LLC inoculation. n = 5 mice per group. BL, baseline; CSF, cerebrospinal fluid. Data displayed represent the mean ± SEM, and are analyzed with two-tailed Student’s t-test, * *P*<0.05.

**Table S1. General information of enrolled cancer patients**

| **Items** | **NPD1-low (n = 40)** | **NPD1-high (n = 39)** | ***P*-value** |
| --- | --- | --- | --- |
| Age (y) | 63 ± 7 | 62 ± 10 | 0.633 |
| Sex (n/%) |  |  |  |
| Male | 20 (50) | 21 (53.8) | 0.732 |
| Female | 20 (50) | 18 (46.2) |  |
| BMI | 24.76 ± 3.58 | 24.66 ± 3.14 | 0.899 |
| Primary Cancer (n/%) |  |  |  |
| Lung cancer | 27 (67.5) | 26 (66.7) | 0.937 |
| Breast cancer | 13 (32.5) | 13 (33.3) |  |
| No. of bone lesions |  |  |  |
| ≤5 | 19 (47.5) | 20 (51.3) | 0.737 |
| >5 | 21 (52.5) | 19 (48.7) |  |
| Surgical treatment |  |  |  |
| Yes | 27 (67.5) | 26 (66.7) | 0.937 |
| No | 13 (32.5) | 13 (33.3) |  |

**Table S2. General information of mice used in the current study**

| **Figures** | **No.** | **Sex** | **Age (weeks)** | **Type of mice** |
| --- | --- | --- | --- | --- |
| 1 B-D | 30 | 15 male, 15 female | 8-10 | WT |
| 1 E | 30 | 15 male, 15 female | 8-10 | WT |
| 2 B-H | 32 | 16 male, 16 female | 8-10 | WT |
| 3 B-G | 24 | 12 male, 12 female | 8-10 | WT |
| 4 B-G | 24 | 16 male, 8 female | 8-10 | WT, *Gpr37^−/−^* |
| 5 A-I | 8 | male | 8-10 | WT |
| 6 B-E | 15 | male | 7-8 | WT |
| 6 F-I | 15 | male | 7-8 | *Gpr37^−/−^* |
| 7 B-D | 10 | male | 8-10 | WT |
| 7 G-H | 12 | male | 8-10 | WT, *Gpr37^−/−^* |
| 8 H | 1 | female | 8-10 | *Arrb2* CKO |
| 8 I-J | 20 | female | 8-10 | WT, *Arrb2* CKO |
| 9 A | 1 | male | 8-10 | WT |
| 9 B-E | 12 | male | 5-6 | WT |
| 9 F-I | 12 | male | 5-6 | *Gpr37^−/−^* |
| S1 A-E | 15 | male | 8-10 | WT |
| S1 F-J | 15 | female | 8-10 | WT |
| S2 B-D | 18 | male | 8-10 | WT |
| S3 B-G | 24 | female | 8-10 | WT |
| S4 B-G | 24 | 16 male, 8 female | 8-10 | WT, *Gpr37^−/−^* |
| S5 | 10 | male | 8-10 | WT, *Gpr37^−/−^* |
| S6 | 20 | male | 8-10 | WT, *Gpr37^−/−^* |
| S7 A-D | 18 | male | 8-10 | WT |
| S7 E-F | 3 | male | 8-10 | WT |
| S7 G-J | 8 | male | 8-10 | WT, *Gpr37^−/−^* |
| S8 C-D | 6 | male | 8-10 | WT, *Gpr37^−/−^* |
| S10 | 15 | male | 8-10 | WT |
